# Up-regulation of CB_1_ cannabinoid receptors located at glutamatergic terminals in the medial prefrontal cortex of the obese Zucker rat

**DOI:** 10.3389/fnana.2022.1004702

**Published:** 2022-10-18

**Authors:** Leyre Echeazarra, Sergio Barrondo, Gontzal García del Caño, Itziar Bonilla-Del Río, Jon Egaña-Huguet, Nagore Puente, Xabier Aretxabala, Mario Montaña, Maider López de Jesús, Imanol González-Burguera, Miquel Saumell-Esnaola, María Aránzazu Goicolea, Pedro Grandes, Joan Sallés

**Affiliations:** ^1^Department of Physiology, Faculty of Pharmacy, University of the Basque Country (UPV/EHU), Vitoria-Gasteiz, Spain; ^2^Bioaraba, Dispositivos Móviles para el Control de Enfermedades Crónicas, Vitoria-Gasteiz, Spain; ^3^Department of Pharmacology, Faculty of Pharmacy, University of the Basque Country (UPV/EHU), Vitoria-Gasteiz, Spain; ^4^Centro de Investigación Biomédica en Red de Salud Mental, Madrid, Spain; ^5^Bioaraba, Neurofarmacología Celular y Molecular, Vitoria-Gasteiz, Spain; ^6^Department of Neurosciences, Faculty of Pharmacy, University of the Basque Country (UPV/EHU), Vitoria-Gasteiz, Spain; ^7^Department of Neurosciences, Faculty of Medicine and Nursing, University of the Basque Country (UPV/EHU), Leioa, Spain; ^8^Achucarro Basque Center for Neuroscience, Science Park of the University of the Basque Country (UPV/EHU), Leioa, Spain; ^9^Department of Analytical Chemistry, Faculty of Pharmacy, University of the Basque Country (UPV/EHU), Vitoria-Gasteiz, Spain; ^10^Division of Medical Sciences, University of Victoria, Victoria, BC, Canada

**Keywords:** obese Zucker rat, CB1 receptor, G_*i/o*_ proteins, 2-AG levels, medial prefrontal cortex, glutamatergic terminals, excitatory synapses, long-term potentiation

## Abstract

The present study describes a detailed neuroanatomical distribution map of the cannabinoid type 1 (CB_1_) receptor, along with the biochemical characterization of the expression and functional coupling to their cognate G_*i/o*_ proteins in the medial prefrontal cortex (mPCx) of the obese Zucker rats. The CB_1_ receptor density was higher in the prelimbic (PL) and infralimbic (IL) subregions of the mPCx of obese Zucker rats relative to their lean littermates which was associated with a higher percentage of CB_1_ receptor immunopositive excitatory presynaptic terminals in PL and IL. Also, a higher expression of CB_1_ receptors and WIN55,212-2-stimulated [^35^S]GTPγS binding was observed in the mPCx but not in the neocortex (NCx) and hippocampus of obese rats. Low-frequency stimulation in layers II/III of the mPCx induced CB_1_ receptor-dependent long-term synaptic plasticity in IL of area obese Zucker but not lean rats. Overall, the elevated 2-AG levels, up-regulation of CB_1_ receptors, and increased agonist-stimulated [^35^S]GTPγS binding strongly suggest that hyperactivity of the endocannabinoid signaling takes place at the glutamatergic terminals of the mPCx in the obese Zucker rat. These findings could endorse the importance of the CB_1_ receptors located in the mPCx in the development of obesity in Zucker rats.

## Introduction

The genetically obese Zucker (fa/fa) rat is one of the available animal models used to study the genetic and neurochemical factors that contribute to food consumption and hyperphagia (Guillaume-Gentil et al., 1990; di Marzo et al., 2001; Simler et al., 2006; Rasmussen et al., 2010). The behavioral phenotype of the obese Zucker rat, which develops innately due to a genetically determined leptin receptor defect that results in leptin resistance and obesity, is associated with increased levels of appetite-stimulating signals including endocannabinoids (di Marzo et al., 2001). Moreover, during adulthood of the obese Zucker rat, an increased expression of cannabinoid type 1 (CB_1_) receptors occurs innately in several cortical areas (Thanos et al., 2008b; Zarate et al., 2008a) that are remarkably related to feeding behavior. Interestingly, obese Zucker rats exhibit a higher sensitivity to cannabinoid drugs than lean rats (Vickers et al., 2003; Rasmussen and Huskinson, 2008; Serrano et al., 2008; Smith and Rasmussen, 2010; Boomhower et al., 2013; Buckley and Rasmussen, 2014). Furthermore, at both low and high response requirement settings for food consumption, rimonabant, and other CB_1_ receptor antagonists reduce food intake (Vickers et al., 2003) and attenuate the reinforcing properties of palatable food (Rasmussen and Huskinson, 2008; Rasmussen et al., 2010), suppressing food-reinforced behavior (Rasmussen et al., 2012; Buckley and Rasmussen, 2014) and increasing sensitivity to response requirements for sucrose (Rasmussen and Huskinson, 2008; Rasmussen et al., 2012). Finally, consistent with studies showing that CB_1_-specific drugs affect impulsivity-related processes in rats (Pattij et al., 2007) and humans (McDonald et al., 2003), the obese Zucker rats behave more impulsively than their lean littermates in food consumption (Boomhower et al., 2013). In view of previous reports that the proper functioning of the rodent medial prefrontal cortex (mPCx), a brain region associated with decision making (Royall et al., 2002), depends on the balance between excitatory and inhibitory synaptic transmission (E/I balance) (Yizhar et al., 2011), it is conceivable that changes in the net impact of CB_1_ receptor-mediated effects on E/I balance may contribute to the behavioral deficits observed in the obese Zucker rat. In this sense, it is still unknown to what extent the up-regulation of brain cortical CB_1_ receptors described in previous reports (Thanos et al., 2008b; Zarate et al., 2008a) is associated with neurons of the excitatory or inhibitory type in the mPCx. Furthermore, although our previous studies in this model showed increased CB_1_ receptor expression in the frontal cortex and related limbic areas of the obese Zucker rat (Zarate et al., 2008a,b), CB_1_ immunoreactivity was distributed not only in its canonical location at axon terminals but also in controversial somatodendritic locations (Zarate et al., 2008a). We discuss here this discrepancy on the basis of a recent study from our laboratory that unravels technical issues relevant to the specific immunohistochemical detection of the CB_1_ receptor in the rodent brain (Echeazarra et al., 2021) and provides a framework to interpret past and future results derived from the use of different anti-CB_1_ receptor antibodies.

With this evidence in mind, we reasoned that the obese Zucker rat, as a behavioral phenotype of impulsive approach to food, provides an excellent opportunity to further assess changes in the key elements of the endocannabinoid system in the mPCx, a key brain region for the cognitive control of substance use behavior (Volkow et al., 2003). Here, we compared the expression levels of CB_1_ receptors and of the members of the Gα_*i/o*_ family of G protein subunits, as well as analyzed CB_1_ receptor-Gα_*i/o*_ functional coupling in the mPCx of obese and lean Zucker rats and extended the analyses to neocortex (NCx) and hippocampus to verify whether possible changes in the parameters analyzed are region-specific or, on the contrary, a general phenomenon. Simultaneously, we determined the levels of 2-arachidonoylglycerol (2-AG), as the most abundant endogenous ligand involved in retrograde cannabinoid signaling at synapses (Stella et al., 1997; Gao et al., 2010; Tanimura et al., 2010; Yoshino et al., 2011), to gain insights in its brain distribution pattern in relation with the pattern of CB_1_ receptor expression, as previously reported in male Wistar strain rats (Bisogno et al., 1999). In fact, it has been reported an increased accumulation of 2-AG in the hypothalamus of obese Zucker rats with respect to their lean controls (di Marzo et al., 2001). Our results add experimental support to the hypothesis that the obese Zucker rat may represent a preclinical model of vulnerability to obesity, conceivably linked with an altered endocannabinoid system-dependent E/I balance at the mPCx.

## Materials and methods

### Animals

**FIGURE 1:**
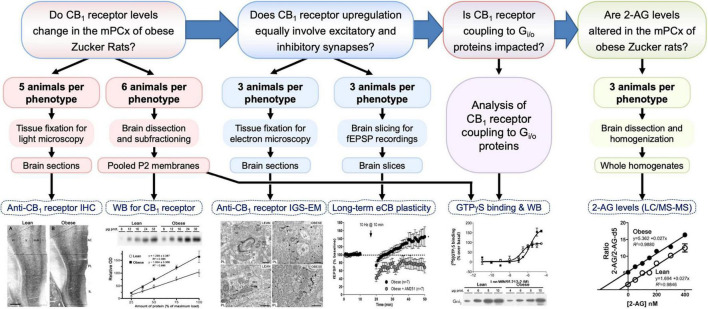
Diagram summarizing the workflow, sample size and processing, and experimental approaches to answer the four main questions (framed boxes at top) posed in this study. 2-AG, 2-arachidonoylglycerol; eCB, endocannabinoid; fEPSP, field excitatory postsynaptic potential response; IGS-EM, immunogold-silver staining for electron microscopy; IHC, immunohistochemistry; LC/MS-MS, liquid chromatography and mass spectrometry; mPCx, medial prefrontal cortex; WB, Western blot.

Forty 12-week-old male lean and obese Zucker rats were used in this study. Sixteen animals (8 per genotype) were used for immunohistochemistry and electronic microscopy, 12 (6 per genotype) were used for both Western blot analysis and functional binding studies, 6 (3 per genotype) were used for electrophysiological studies, and the remaining 6 (3 per genotype) for liquid chromatography and mass spectrometry (LC/MS-MS) analysis (Figure 1). Animals were purchased from Charles River Laboratories España S.A. (Barcelona, Spain), housed in a controlled environment (22 ± 2°C: 12 h light-dark cycle) with food and water provided *ad libitum*, and allowed to acclimate for at least 2 weeks before culling between 10:00 and 12:00 AM. Animal handling was carried out in accordance with the EU Directive 2010/63/EU and experimental procedures were approved by the Ethics/Animal Committee of the University of the Basque Country (CEBA/199/2011/GARCIA DEL CAÑO). All efforts were made to minimize animal suffering and to reduce the number of animals used.

### Drugs and chemicals

Guanosine 5′-o-(3-[^35^S]thio-triphosphate) ([^35^S]GTPγS, 1000–1400 Ci/mmol) was purchased from Perkin-Elmer (Barcelona, Spain). Protease inhibitor cocktail (phenylmethylsulfonyl fluoride -PMSF- and iodoacetamide), GDP, GTP, GTPγS, and WIN 55,212-2 were purchased from Sigma Chemical (St. Louis, MO, USA). Picrotoxin (PTX) and AM251 were purchased from Tocris Bioscience (Bristol, UK). For the endocannabinoid determinations, 2-AG and 1-AG, and their deuterated analog, 2-AG-d_5_ and 1-AG-d_5_, were obtained from Cayman Chemicals. Water (H_2_O), acetonitrile, formic acid, ethylacetate, and hexane (all of Fluka LC-MS grade) were obtained from Sigma Aldrich.

### Immunohistochemistry for light microscopy and preembedding immunogold for electron microscopy

Rats were anesthetized with an overdose of choral hydrate (1 g/kg, i.p.; Panreac Química S.A., Castellar del Vallés, Barcelona, Spain) and perfused transcardially at a constant flow of 30 ml/min (Heidolph Instruments GmbH & Co. KG, Pumpdrive PD 5106, Schwabach, Germany) for 1 min with 0.1 M phosphate-buffered saline (PBS, pH 7.4), followed by 5 min perfusion with fixative solution made up of either 4% paraformaldehyde in 0.1 M phosphate buffer (PB pH 7.4), for light microscopy, or 4% paraformaldehyde, 0.2% picric acid and 0.1% glutaraldehyde in PB, for electron microscopy. Thereafter, brains were removed and post-fixed overnight in the same fixative for 4 h. Brains intended to obtain sections for light microscopy were immersed in a cryoprotective solution (30% sucrose in 0.1 M PB, pH 7.4) for 48 h.

Sections for immunohistochemical staining for light microscopy were cryosectioned at 40 μm using a microtome (Leitz-Wetzlar 1310, Wetzlar, Germany) equipped with a temperature sensor (5MP BFS-Physitemp Controller, Clifton, NJ, USA) and processed for immunohistochemical staining as previously described (Montaña et al., 2012; Echeazarra et al., 2021) using an affinity-purified goat polyclonal antibody raised against the C-terminal 31 amino acids (443–473) of the mouse CB_1_ receptor (CB1-Go-Af450; Frontier Science Co. Ltd, Hokkaido, Japan) (Table 1). For electron microscopy, 50 μm-thick vibratome sections of the mPCx were collected in 0.1 M phosphate buffer (pH 7.4) at room temperature and processed for pre-embedding silver-intensified immunogold using the CB1-Go-Af450 antibody (Table 1) as previously described (Bonilla-Del Río et al., 2021). Details of the immunohistochemical and pre-embedding silver-intensified immunogold methods are provided in Supplementary Methods.

**TABLE 1:** Primary antibodies used.

Target	Dilution (ng/mL)			Host and clonality	Purity and isotype	Immunizing antigen	Source, catalog
	IHC	IGS-EM	WB				
CB_1_ receptor	1000	2000	200	Goat polyclonal	Immunogen affinity-purified IgG	31 amino acids at the C-terminus of mouse CB_1_	Frontier Institute Co., Ltd., CB1-Go-Af450
Gα _*o*_ subunit	—	—	40	Rabbit polyclonal	Protein A/G affinity-purified IgG	Peptide within a highly divergent domain of rat Gα_*o*_	Santa Cruz Biotech. Inc., sc-387
Gα _*i1*_ subunit	—	—	40	Rabbit polyclonal	Protein A/G affinity-purified IgG	Peptide within a highly divergent domain of rat Gα_*i1*_	Santa Cruz Biotech. Inc., sc-391
Gα _*i2*_ subunit	—	—	200	Rabbit polyclonal	Protein A/G affinity-purified IgG	Peptide within a highly divergent domain of rat Gα_*i2*_	Santa Cruz Biotech. Inc., sc-7276
Gα _*i3*_ subunit	—	—	5	Rabbit polyclonal	Protein A/G affinity-purified IgG	Peptide mapping at the C-terminus of rat Gα_*i3*_	Santa Cruz Biotech. Inc., sc-262
PLC-β _1_	—	—	32	Mouse monoclonal	Protein A/G affinity-purified IgG_1_	Amino acids 4–159 of rat PLC-β_1_	BD Transduction Labs., 610924

IHC, immunohistochemistry; IGS-EM, immunogold-silver staining for electron microscopy; WB, Western blot. Antibody manufacturers: Santa Cruz Biotechnology, Santa Cruz, CA, USA; BD Transduction Laboratories, San Diego, CA, USA; Frontier Institute Co., Ltd., Hokkaido, Japan

### Light microscopy imaging and semiquantitative analysis of the CB_1_-immunostaining

Brain sections processed for immunohistochemistry were examined with an Olympus BX50F light microscope (Olympus, Tokyo, Japan) connected to a high-resolution digital camera (Olympus and Soft Imaging Systems, Tokyo, Japan). All microscope images were digitized with a grayscale resolution of 16 bits/pixel using CellA software for image acquisition (Olympus and Soft Imaging Systems, Tokyo, Japan) and saved in TIFF format. Microscope images used for illustration were exported as 8-bit grayscale TIFF files and compiled and labeled using Adobe Photoshop CS3 software (San Jose, CA, USA).

Images used for semiquantitative analysis of the optical density (OD) of CB_1_-immunostaining in lean and obese Zucker rats were acquired under identical conditions (exposure time and intensity of illumination) with a 10×objective and a grayscale resolution of 16 bits/pixel. Measurements were performed in several areas of the cortex and hippocampal formation of lean and obese Zucker rats on microscope images acquired. Within the cortex, prelimbic (PL) and infralimbic (IL) subregions of mPCx, primary motor cortex (MOp), and anterior cingulate cortex (AC) were chosen for analysis. Within the hippocampal formation, analyses were performed in *dentate gyrus* (DG) and fields CA1, CA2, and CA3 of the Ammon’s horn. Quantification was carried out using the measure function of ImageJ image analysis software (ImageJ, NIH, Bethesda, MD, USA) (details are given in Supplementary Methods and Supplementary Figures 1A,B).

### Electron microscopy imaging and quantification of CB_1_-immunogold particles

Ultrathin sections of 50 nm were collected on mesh nickel grids, stained with 2.5% lead citrate for 20 min, and examined in a Philips EM208S electron microscope. Tissue preparations were photographed using a digital camera coupled to the electron microscope. Images used for illustration were exported as 8-bit grayscale TIFF files and compiled and labeled using Adobe Photoshop CS3 software (San Jose, CA, USA).

Sampling was performed accurately and in the same way for all the animals studied. Before the ultra-thin sectioning, immunogold-labeled sections were visualized under the light microscope in order to select portions of the IL and PL areas displaying good and reproducible CB_1_ receptor immunolabelling. Electron micrographs were taken at 22,000 × using a Digital Morada Camera from Olympus. Analyses were performed on the first five 50 nm-ultrathin sections obtained within the first 1.5 μm from the surface of the tissue block. To determine the proportion of CB_1_ receptor positive terminals, positive labeling was considered when at least one CB_1_ receptor immunoparticle was within ∼30 nm from the membrane of the specific compartment under study. The excitatory and inhibitory synapses were identified on the basis of their ultrastructural features. Excitatory synapses are asymmetrical with postsynaptic densities and presynaptic axon terminals containing abundant, clear, and spherical synaptic vesicles. Inhibitory synapses are symmetrical with slender postsynaptic membranes and axon terminals containing pleomorphic synaptic vesicles. To determine the percentages of excitatory and inhibitory terminals containing CB_1_ receptor particles and the CB_1_ receptor density (particles/μm membrane) in both types of terminals electron microscope images were analyzed on ImageJ. Statistics were done on GraphPad Prism 8.

### Slice preparation and extracellular field recordings

Lean and obese Zucker rats were anesthetized with isoflurane (2–4%) and brains were rapidly removed and placed in a sucrose-based solution at 4°C that contained (in mM): 87 NaCl, 75 sucrose, 25 glucose, 7 MgCl_2_, 2.5 KCl, 0.5 CaCl_2_, and 1.25 NaH_2_PO_4_. Coronal vibratome sections (300 μm thick, Leica Microsistemas S.L.U.) were collected, recovered at 32–35°C before being placed in the recording chamber, and superfused (2 ml/min) with artificial cerebrospinal fluid (aCSF) containing (in mM): 130 NaCl, 11 glucose, 1.2 MgCl_2_, 2.5 KCl, 2.4 CaCl_2_, 1.2 NaH_2_PO_4_, and 23 NaHCO_3_, equilibrated with 95% O_2_/5% CO_2_. All experiments were carried out at 32–35°C. Picrotoxin (PTX; 100 μM, Tocris Bioscience, Bristol, UK) was added to the aCSF to block GABA_*A*_ receptors.

For extracellular field recordings, a glass-recording pipette was filled with aCSF. The stimulation and recording electrodes (borosilicate glass capillaries, Harvard apparatus UK capillaries 30–0062 GC100T-10) were placed in IL layers II/III of the mPCx. To evoke field excitatory postsynaptic potential responses (fEPSPs), repetitive control stimuli were delivered at 0.1 Hz (Stimulus Isolater ISU 165, Cibertec, Spain; controlled by aMaster-8, A.M.P.I.). An Axopatch-200B (Axon Instruments/Molecular Devices, Union City, CA, USA) was used to record the data filtered at1–2 kHz, digitized at 5 kHz on a DigiData 1440A interface, collected on a PC using Clampex 10.0 and analyzed using Clampfit 10.0 (all obtained from Axon Instruments/Molecular Devices, Union City, CA, USA). At the start of each experiment, an input-output curve was constructed. Stimulation intensity was selected for baseline measurements that yielded between 40 and 60% of the maximal amplitude response. To induce eCB-eLTD of glutamatergic inputs, a low-frequency stimulation (LFS, 10 min at 10 Hz) protocol was applied following the recording of a steady baseline as described previously (Puente et al., 2011; Peñasco et al., 2020). The magnitude of the fEPSP area for eCB-eLTD was calculated as the percentage change between the baseline area (averaged excitatory responses for 10 min before LFS) and the last 10 min of stable responses, recorded 30 min after the end of the LFS. At least three rats were used for each experimental condition.

### Tissue sampling for western blot, [^35^S]GTPγS binding, and 2-AG measurements

Rats were anesthetized with an overdose of choral hydrate (1 g/kg, i.p.; Panreac Química S.A.) and sacrificed animals by decapitation. Immediately after culling, brains were removed and cortical samples were dissected on an ice-chilled glass plate under a stereomicroscope (Nikon, SMZ800, Nikon Instruments Europe, B.V Amstelveen, Netherlands), using the midline, the corpus callosum and the rhinal fissure as anatomical landmarks (Supplementary Figure 2). Immediately after dissection, cortical and hippocampal samples were frozen in isopentane pre-cooled to –80°C. Samples of mPCx and NCx from 12 animals (six lean and six obese Zucker rats) were used for Western blot analysis and [^35^S]GTPγS binding assays. Western blot was also performed in hippocampal samples from six lean and six obese Zucker rats. Samples of prefrontal, anterior and posterior NCx from six animals (three lean and three obese Zucker rats) were used for 2-AG measurements by LC/MS-MS analysis. Samples of mPCx, NCx, and hippocampus intended for Western blot analysis and [^35^S]GTPγS binding assays were pooled from groups of six animals of the same genotype, thus giving two pools per cortical region and genotype. Samples intended for LC/MS-MS were treated individually.

### Tissue fractionation for western blot and [^35^S]GTPγS binding

Frozen tissue samples were thawed in ice-cold homogenization buffer: 20 mM Tris-HCl, 0.32 M sucrose, 1 mM ethylene glycol bis (2-aminoethyl ether) tetraacetic acid (EGTA), and protease inhibitors (1 mM phenylmethylsulfonyl fluoride -PMSF- and 0.5 mM iodoacetamide), pH 7.5. Tissue was homogenized in 20 volumes of the same buffer and centrifuged at 1,100 × *g* for 10 min at 4°C. The pellet containing nuclei and cell debris was discarded and the supernatant was centrifuged at 14,000 × *g* for 30 min at 4°C. The resulting pellet was re-suspended in the same volume, distributed in 1 ml aliquot into 1.5 ml Eppendorf tubes, and centrifuged at 40,000 × *g* for 10 min. The supernatant was discarded and the aliquots of the final washed pellets of crude membranes (P2) were stored at –80°C until use. The protein content in P2 pellets and the protein concentration in Cyt fraction were determined by the Bradford method with the Bio-Rad dye reagent (Hercules, CA, USA) using bovine γ-globulin as standard.

### Western blotting and linear regression analysis of protein expression

Western blot analysis was performed as previously described (Montaña et al., 2012; Saumell-Esnaola et al., 2021) with minor modifications. CB_1_ cannabinoid receptor, G_*i/o*_ α-subunits, and phospholipase C-β_1_ (PLC-β_1_) were detected by immunoblotting with the following antibodies: goat polyclonal anti-CB_1_ receptor (CB1-Go-Af450; Frontier Science Co. Ltd, Hokkaido, Japan), rabbit polyclonal anti-Gα_*o*_ (sc-387; Santa Cruz Biotechnology, Santa Cruz, CA, USA), rabbit anti-Gα_*i*1_ (sc-391; Santa Cruz Biotechnology), rabbit polyclonal anti-Gα_*i*2_ (sc-7276; Santa Cruz Biotechnology), rabbit polyclonal anti-Gα_*i*3_ (sc-262; Santa Cruz Biotechnology) and mouse monoclonal anti-PLC-β_1_ (BD Transduction Laboratories, San Diego, CA, USA) (Table 1). Details of the Western blot procedure are given in Supplementary Methods.

Immunoreactive signals produced by increasing amounts of total P2 protein were analyzed by linear regression. First, the linear range for detection of the CB_1_ receptor, Gα_*o*_, and Gα_*i*1–3_ subunits, and PLC-β_1_ proteins was generated by immunoblotting increasing amounts (total protein) of P2 fraction, followed by densitometric analysis using the gel analysis tool of ImageJ software. For each Western blot assay, two samples from each genotype (each sample corresponding to a pool from three rats) were thawed and denatured. Increasing amounts of total protein from sample pools of lean and obese Zucker rats were resolved and processed in parallel for immunoblot. Thus, densitometric analysis of specific immunoreactive bands provided one raw integrated OD density value per protein load point and sample pool.

### WIN55,212-2-stimulated [^35^S]GTPγS specific binding to brain membranes

The [^35^S]GTPγS binding assays were performed following the procedure described elsewhere for mice and human brain membranes (Saumell-Esnaola et al., 2021). Briefly, brain cortical and hippocampal membranes were thawed, and incubated at 30°C for 2 h in [^35^S]GTPγS-incubation buffer (0.5 nM [^35^S]GTPγS, 1 mM EGTA, 3 mM MgCl_2_, 100 mM NaCl, 0,2 mM DTT, 50 μM GDP, and 50 mM Tris-HCl, pH 7.4). The cannabinoid CB_1_ receptor agonist WIN 55,212-2 (10^–9^–10^–5^ M, eight concentrations) was added to determine receptor-stimulated [^35^S]GTPγS binding. Non-specific binding was defined in the presence of 10 μM unlabeled GTPγS. Basal binding was assumed to be the specific [^35^S]GTPγS binding in the absence of agonist. The reactions were terminated by rapid vacuum and filtration through Whatman GF/C glass fiber filters and the remaining bound radioactivity was measured by liquid scintillation spectrometry as described above.

### Measurement of cortical brain endogenous 2-AG levels by liquid chromatography and mass spectrometry

Samples (95–120 mg wet weight) were weighed into borosilicate tubes containing 2 ml ice-cold 0.1 M formic acid and were homogenized with the aid of a 5 mm-steel ball using the Digital Sonifier (Model S250 Branson, USA) for 1 cycle of 10 s at 10% amplitude. Aliquots (50 μl) of the homogenate were placed into silanized microcentrifuge tubes containing ice-cold 0.1 M formic acid and were spiked with 20 μl acetonitrile containing the internal standards (deuterated 2-AG-d_5_ and deuterated 1-AG-d_5_, final concentration 100 nM) and with 10 μl of the appropriate concentration of 2-AG in its natural form, to give a final volume of 500 μl. Ethylacetate/hexane (1,000 μl; 9:1, v/v) was added to extract the cortical homogenate, again with the aid of the Digital Sonifier for 1 cycle of 10 s at 10% amplitude. Then, the tubes were centrifuged for 10 min at 10,000 × *g* and 4°C, and the upper (organic) phase was removed, evaporated to dryness under a gentle stream of nitrogen at 37°C, and re-dissolved in 500 μl acetonitrile.

Analyses were performed as previously described (Schulte et al., 2012) on an LC-MS/MS system based on Agilent technologies (Wilmington) consisting of a 6410 Triple Quad mass spectrometer equipped with an electrospray ionization source operating in positive ion mode, and a 1200-series binary pump system. 2-AG was separated with a Phenomenex Luna 2.5 μm C18(2)-HST column, 100 × 2 mm, combined with a Security Guard pre-column (C18, 4 × 2 mm; Phenomenex) with solvents A (0.1% formic acid in 20:80 acetonitrile/water, v/v) and B (0.1% formic acid in acetonitrile), using the following gradient: 55–90% B (0–2 min), then held at 90% B (2–7.5 min) and re-equilibrated at 55% B (7.5–10 min). The column temperature was 25°C, the flow rate was 0.3 mL/min, the injection volume was 10 μL and the needle was rinsed for 60 s using a flushport with Water/Acetonitrile (80:20) as the eluent. The electrospray ionization interface was operated using nitrogen as a nebulizer and desolvation gas, and using the following settings: temperature 350°C, nebulizer pressure 40 psi, and capillary voltage + 4,800 V. The following precursor-to-product ion transitions were used for multiple-reaction monitoring (MRM): 2-AG and 1-AG m/z 379.4→287; 2-AG-d_5_ and 1-AG-d_5_ m/z 384→287. Dwell times were 20 ms; pause between MRM transitions was 5 ms. Data acquisition and analysis were performed using MassHunter Software.

The determination of the endogenous 2-AG levels by LC-MS/MS was carried out as described previously (Schulte et al., 2012) using a strategy of isotope dilution combined with standard addition techniques developed in our laboratory (García Del Caño et al., 2015) in order to get an accurate quantification of the endogenous levels of 2-AG. Thus, we spiked into each aliquot of the brain cortical homogenate the same amount of the internal standard solution (2-AG-d_5_), while 2-AG in its natural form was spiked with increased amounts (standard additions: 50, 100, 150, 200, 300, and 400 nM) into the series of subsamples tubes except the first one. In these conditions, it is possible to find a linear relationship with an excellent correlation coefficient between the concentration of 2-AG spiked to the homogenate and the ratio of the obtained areas for 2-AG and 2-AG-d_5_. Finally, the intercept on the x-axis provides the endogenous level of 2-AG in the aliquot of the brain cortical homogenate. Since 2-AG undergoes rapid isomerization to 1-AG under common experimental conditions (Zoerner et al., 2011), all the samples were also spiked with the internal standard 1-AG-d_5_ (100 nM), which allowed us to analyze to what extent the 1-AG isomer contributes to the analytical measurement. In our hands, the levels of 1-AG usually represent 5% of 2-AG and were not included in the total 2-AG amounts.

### Statistical analysis

The statistical analysis was performed using GraphPad Prism 8 software (GraphPad Software Inc., San Diego, CA, USA). All values are given as mean ± standard error of the mean (SEM) unless otherwise indicated. The significance level was set at *p* < 0.05 for all comparisons. Differences between lean and obese Zucker rats in relative OD for CB_1_-immunostaining were analyzed by two-way analysis of variance (ANOVA), with genotype (lean and obese) and cortical area (MOp, mPCx, AC, PL, and IL), or genotype (lean and obese) and hippocampal region (CA1, CA2, CA3, DG) as the main factors, followed by Bonferroni *post hoc* test. Data obtained by immunogold-silver staining and electron microscopy (percentages of excitatory and inhibitory terminals containing CB_1_ receptor particles and the CB_1_ receptor density) were analyzed using parametric or non-parametric two-tailed Student’s *t*-test or one-way ANOVA with Bonferroni *post hoc* analysis. The potential variability between rats of the same group was statistically analyzed, finding no differences between them, so all data from each condition were pooled. Electrophysiological data were first analyzed by Shapiro–Wilk and Kolmogorov–Smirnov for normality. In general, statistical significance between conditions (baseline *versus* after drug or stimulation protocol or both) was tested using parametric (two-tailed Student’s *t*-test) or non-parametric (Mann–Whitney test). To analyze OD density values of immunoreactive bands produced by increasing amounts of total protein by Western blot, the integrated OD values obtained from the bands at the maximum amount of protein from samples of lean Zucker rats were averaged and taken as a reference to calculate the percentage OD of each band’s integrated OD. Best-fit lines were generated using GraphPad Prism and the statistical significance of the difference between slopes was analyzed by an *F*-test. Individual WIN 55,212-2 concentration-response curves were fitted by non-linear regression to the four-parameter Hill equation, using Graph Pad Prism 8. The statistical significance of differences between the means of the parameter estimates was evaluated by Student’s *t*-test. Because the affinity constants are obtained experimentally to have a log normal distribution, *EC*_50_ values are logarithmically transformed for statistical analysis (Christopoulos, 1998). The significance of differences between mean values of 2-AG levels determined by LC/MS-MS was analyzed by unpaired two-tailed Student’s *t*-test.

## Results

### CB_1_ receptor expression is selectively up-regulated in infralimbic and prelimbic areas of the medial prefrontal cortex of obese Zucker rats

**FIGURE 2:**
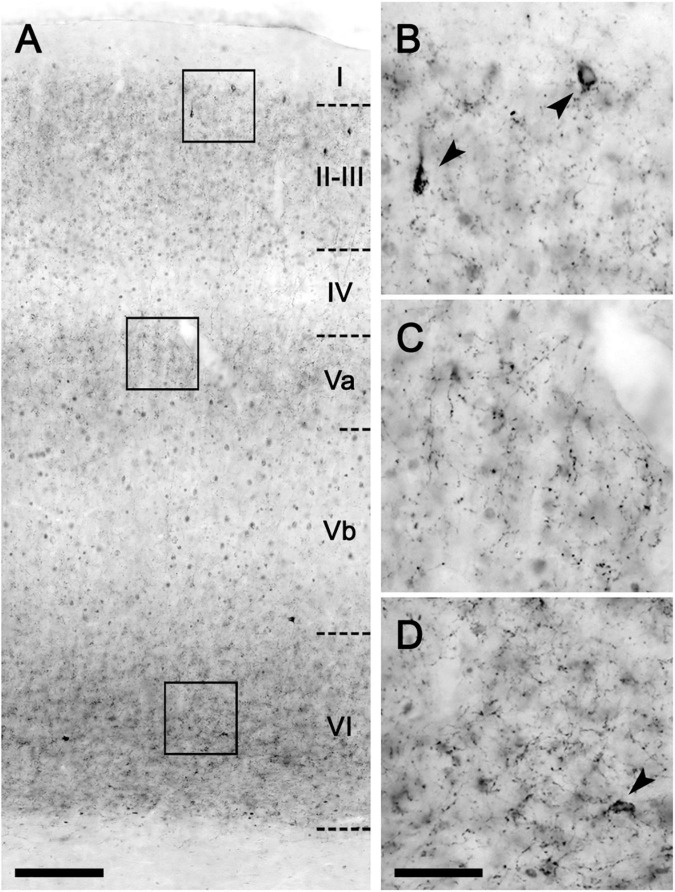
CB_1_-immunohistochemical staining pattern in the neocortex of the lean Zucker rat. **(A)** Low magnification micrograph of the primary motor neocortical area showing the layer distribution of CB_1_-immunostaining. **(B–D)** Higher magnification images of areas framed in A to illustrate the immunostaining pattern in layers II **(B)**, Va **(C),** and VI **(D)**. Arrowheads indicate CB_1_-immunopositive interneurons. Scale bars = 100 μm in **(A)**; 25 μm in **(D)** (applies to **B–D**).

**FIGURE 3:**
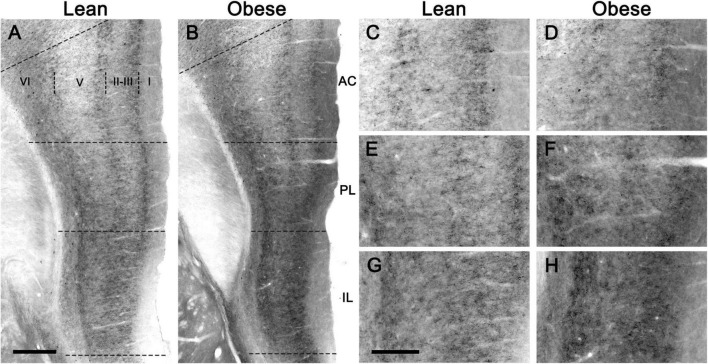
CB_1_-immunostaining in the medial prefrontal cortex of lean and obese Zucker rats. **(A,B)** Low power micrographs showing the distribution of CB_1_-immunostaining in representative coronal sections of the medial prefrontal cortex of a lean **(A)** and an obese **(B)** Zucker rat. **(C–E)** Higher magnification micrographs of the sections shown in **(A,B)**, taken at the level of the anterior cingulate **(C,D)**, prelimbic **(E,F),** and infralimbic **(G,H)** cortices. All images were captured under the same conditions and processed in parallel. AC, anterior cingulate cortex; IL, infralimbic cortex; PL, prelimbic cortex. Scale bars = 200 μm in **(A)** (applies to **A,B**); 400 μm in **(G)** (applies to **C–H**).

**FIGURE 4:**
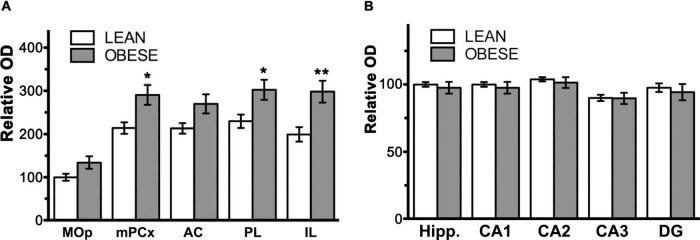
Bar graphs showing the results of semi-quantitative analysis of CB_1_-immunoreactivity in coronal sections through the medial prefrontal cortex and hippocampal formation of lean and obese Zucker rats. **(A)** Results of the analysis in subregions of the medial prefrontal cortex. AC, anterior cingulate cortex; IL, infralimbic cortex; MOp, motor primary cortex; mPCx, medial prefrontal cortex; PL, prelimbic cortex. **(B)** Results of the analysis in subregions of the hippocampal formation. CA1, field CA1 of the Ammon’s horn; CA2, field CA2 of the Ammon’s horn; CA3, field CA3 of the Ammon’s horn; DG, *dentate gyrus*; Hipp., hippocampal formation. Data in **(A,B)** are mean ± SEM; *n* = 5 in each group. Asterisks indicate the significance of the difference between genotypes for a given cortical area (two-way ANOVA followed by *post hoc* Bonferroni’s test). *P < 0.05, **P < 0.01.

**FIGURE 5:**
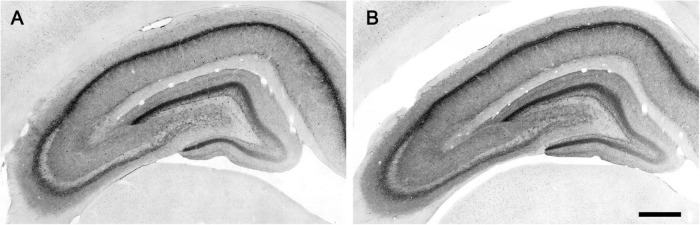
Micrographs of CB_1_-immunostaining in the hippocampal formation of lean **(A)** and obese **(B)** Zucker rats processed in parallel. Scale bar = 500 μm.

The CB_1_ receptor immunostaining pattern in the neocortex (Figure 2) and various subcortical regions (Supplementary Results and Supplementary Figure 3) of the lean Zucker rat matched the described previously in the rat (Egertová and Elphick, 2000; Bodor et al., 2005; Deshmukh et al., 2007; Echeazarra et al., 2021). Thus, in NCx, a layer-specific immunolabelling pattern was clearly observed, being markedly denser in layers II-III, upper part of layer V (Va) and layer VI than in the rest (Figure 2A). CB_1_-immunopositive structures consisted principally of axon-like profiles containing varicosities (Figures 2B–D), along with cell somata, which were sparsely distributed in layers II-III and, to a lesser extent, in layer VI (Figures 2B,D). Similar to the NCx, the distribution of CB_1_-immunostaining within the medial prefrontal cortex showed a marked layered pattern in the lean Zucker rat, although it was stronger compared to neocortical areas. Immunostaining was denser in the upper part of layers II–III and V, and in layer VI than in the rest and increased gradually toward IL subarea (Figures 3A,C,E,G). CB_1_-immunostaining in mPCx of obese Zucker rats was qualitatively the same as that observed in lean rats, but immunoreactivity was considerably more intense in all subareas and layers (Figures 3B,D,F,H). Densitometric analysis of immunostaining in MOp and mPCx subregions followed by two-way ANOVA revealed a significant difference between lean and obese genotypes [*F*_(1_,_40)_ = 34.68, *p* < 0.0001], with no effect of cortical area factor on the observed difference [*F*_(4_,_40)_ = 0.87, *P* = 0.4890]. *Post hoc* Bonferroni’s test revealed a significantly higher CB_1_-immunoreactivity in mPCx, PL, and IL of obese Zucker rats (*p* < 0.05, 0.01, and 0.005, respectively, Figure 4A). A similar analysis was performed in hippocampi of lean and obese Zucker rats. The distribution of CB_1_-immunostaining was virtually identical in all regions of the hippocampal formation of both genotypes with no apparent differences in staining intensity (Figure 5), which was confirmed by densitometric analysis of CB_1_-immunoreactivity in tissue sections (Figure 4B).

**FIGURE 6:**
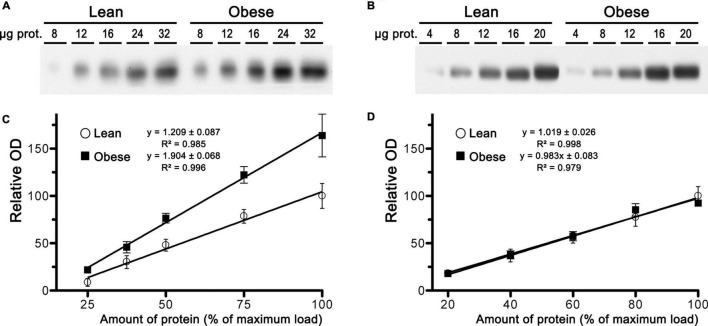
Expression analysis of CB_1_ receptor in samples of crude membranes from medial prefrontal cortex and neocortex of lean and obese Zucker rats. **(A,B)** Representative images of CB_1_ receptor immunoblots using increasing amounts of total protein in crude membranes of mPCx **(A)** and NCx **(B)** from lean and obese Zucker rats. **(C,D)** Linear regression analyses of the relative optical density (OD) measurements of CB_1_-immunoreactivity in crude membranes of mPCx **(C)** and NCx **(D)** from lean (°) and obese (∙) Zucker rats. The correlation coefficients are provided. Data presented are means ± SEM of four independent experiments.

Next, we performed immunoblot analysis of CB_1_ receptor expression in samples of mPCx, NCx, and hippocampus from lean and obese Zucker rats. Linear regression analysis generated by densitometric analysis of CB_1_-immunoreactivity in immunoblots of increasing amounts of crude membranes from lean and obese Zucker rats revealed that expression of the CB_1_ receptor was increased by 57% in mPCx of obese rats in comparison with lean rats [slope differences, *F*_(1_,_36)_ = 10.210, *p* = 0.0029] (Figures 6A,C), whereas no changes were observed in NCx [slope differences, *F*_(1_,_34)_ = 0.071, *p* = 0.7904] (Figures 6B,D) and hippocampus (not shown).

### Increased CB_1_ receptor-labeled excitatory terminals in the medial prefrontal cortex of obese Zucker rats

**FIGURE 7:**
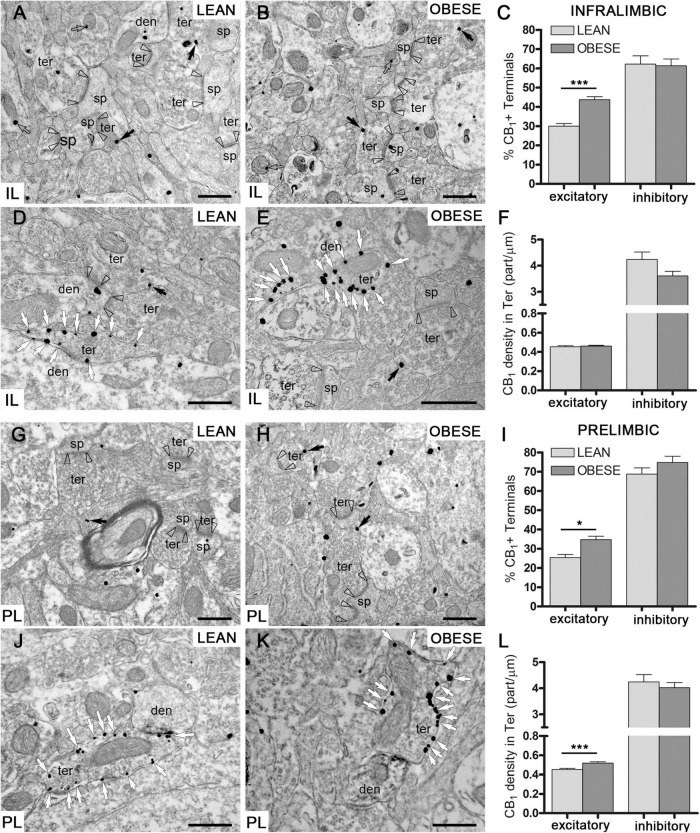
CB_1_ receptor distribution by preembedding immunogold-silver staining for electron microscopy. **(A–F)** CB_1_ receptor particles at excitatory **(A,B)** and inhibitory **(D,E)** synaptic terminals in IL of lean and obese Zucker rats. Percentage of excitatory and inhibitory terminals with CB_1_receptor labeling **(C)** and density of CB_1_ particles at excitatory and inhibitory terminals in IL **(F)**. **(G–L)** CB_1_ receptor labeling at excitatory **(G,H)** and inhibitory **(J,K)** synaptic terminals in PL of lean and obese Zucker rats. Percentage of excitatory and inhibitory terminals containing CB_1_ particles **(I)** and density of CB_1_ labeling at excitatory and inhibitory terminals in PL **(L)**. Arrowheads: excitatory synapses; black arrows: CB_1_ receptor immunoparticles in excitatory terminals; white arrows: CB_1_ receptor immunoparticles in inhibitory terminals; ter, terminal; den, dendrite; sp, dendritic spine. Scale bars = 0.5 μm. Data are mean ± SEM (Mann–Whitney test; *P < 0.05, ***P < 0.0001).

CB_1_-immunopositive particles were localized in presynaptic terminals making symmetric and asymmetric synapses in the IL and PL subregions of the mPCx of lean and obese Zucker rats. In IL, the percentage of excitatory (asymmetric) terminals containing CB_1_ receptor particles (Figures 7A,B) was significantly increased in obese (43.73 ± 1.57%) relative to lean rats (29.95 ± 1.35%) (Mann–Whitney *U* = 31951, *n*_*lean*_ = 308, *n*_*obese*_ = 298, *p* < 0.0001) (Figure 7C). In contrast, the percentage of inhibitory terminals with CB_1_ receptor labeling (Figures 7D,E) was virtually identical between lean (62.19 ± 4.26%) and obese (61.31 ± 3.59%) (Mann–Whitney *U* = 9321, *n*_*lean*_ = 111, *n*_*obese*_ = 171, *P* = 0.7785) (Figure 7C). As expected, these values were considerably higher than the proportion of labeled excitatory terminals (Figure 7C). Furthermore, the density of CB_1_ particles was much higher at inhibitory (lean: 4.24 ± 0.28; obese: 3.61 ± 0.176 particles/μm) than at excitatory terminals (lean: 0.45 ± 0.01; obese: 0.46 ± 0.01 particles/μm). However, no significant differences in CB_1_-receptor density were observed between lean and obese Zucker rats in excitatory (Mann–Whitney *U* = 90938, *n*_*lean*_ = 364, *n*_*obese*_ = 523, *p* = 0.2578) or inhibitory terminals (Mann–Whitney *U* = 7751, *n*_*lean*_ = 107, *n*_*obese*_ = 162, *p* = 0.1427) (Figure 7F).

In PL, the percentage of excitatory terminals containing CB_1_ immunoparticles (Figures 7G,H) was also significantly increased in obese Zucker (34.71 ± 1.77%) relative to lean rats (25.39 ± 1.60%) (Mann–Whitney *U* = 9665, *n*_*lean*_ = 136, *n*_*obese*_ = 168, *P* = 0.0186) (Figure 7I). The percentage of inhibitory terminals with CB_1_ receptor particles was similar in both genotypes (lean: 68.75 ± 3.24%; obese: 74.78 ± 3.20%) (Mann–Whitney *U* = 5502, *n*_*lean*_ = 112, *n*_*obese*_ = 101, *P* = 0.6776), and again considerably higher than the proportion of CB_1_-positive excitatory terminals in lean and obese Zucker rats (Figures 7I–K). Finally, the density of CB_1_ receptor labeling was much higher at inhibitory (lean: 4.24 ± 0.28; obese: 4.02 ± 0.19 particles/μm) than at excitatory terminals (lean: 0.45 ± 0.011; obese: 0.52 ± 0.013 particles/μm), (Figure 7L) however, it was only significantly increased in excitatory terminals of the obese Zucker rats (Mann–Whitney *U* = 8973, *n*_*lean*_ = 140, *n*_*obese*_ = 186, *p* < 0.0001) (Figure 7L).

### Long term synaptic plasticity at excitatory synapses in layers II/III of infralimbic subregion of lean and obese medial prefrontal cortex

**FIGURE 8:**
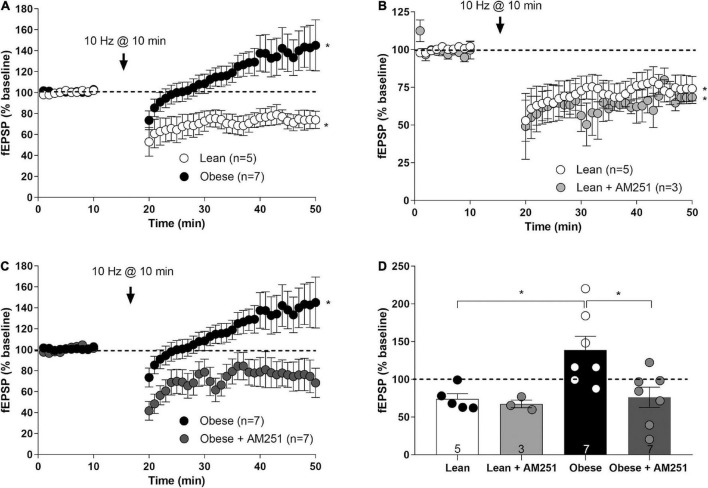
Long-term synaptic plasticity at infralimbic mPCx synapses of the lean and obese Zucker rats. **(A)** Low frequency stimulation (LFS; 10 Hz for 10 min) at the time marked by the *X*-axis break triggers a depression of the fEPSPs (IL-eLTD) in lean rats (white circles; Student’s *t*-test, two tailed, *p < 0.05 *versus* baseline), but a potentiation of the fEPSPs (IL-eLTP) in obese Zucker rats (black circles; Student’s *t*-test, two tailed, *p < 0.05 *versus* baseline). **(B)** Bath perfusion with AM251 (4 μM) in lean rats is ineffective at changing IL-eLTD (light gray circles; Student *t*-test; *P < 0.05 *versus* baseline). **(C)** The slight but significant LTP in obese Zucker rats (black circles) is suppressed by AM251 (4 μM; dark gray circles; Student *t*-test; *P < 0.05 *versus* baseline). **(D)** Summary bar histogram of the experiments performed: lean, lean + AM251 (4 μM), obese, obese + AM251 (4 μM). Student *t*-test; *P < 0.05. Numbers in the bars are individual experiments. Data are expressed as mean ± SEM.

To investigate whether CB_1_ receptor changes in excitatory terminals impact synaptic plasticity, a low-frequency stimulation (LFS, 10 Hz, 10 min) known to elicit long term depression of the excitatory synaptic transmission (eLTD) (Lafourcade et al., 2007; Puente et al., 2011) was applied to mPCx layers II/III of lean and obese Zucker rats. In lean rats, a significant decrease of mean fEPSP amplitude was observed (74.02 ± 6.88% fEPSP relative to baseline; *t* = 3.091, *p* < 0.0149, df = 8) (Figures 8A,D). Unexpectedly, the same protocol triggered excitatory long-term potentiation (eLTP) in obese Zucker rats, as shown by a significant increase in the mean fEPSP (138.8 ± 18.27% fEPSP relative to baseline; Lean *versus* Obese: *t* = 2.858, *P* = 0.0170, df = 10) (Figures 8A,D). Noteworthy, the CB_1_ receptor inverse agonist AM251 (4 μM) did not affect LFS-induced eLTD in lean rats (67.37 ± 5.07% fEPSP relative to baseline; Lean *versus* Lean + AM251: *t* = 0.6729, *p* = 0.5261, df = 6) (Figures 8B,D), whereas suppressed eLTP in obese rats (76.14 ± 13.44% fEPSP relative to baseline; Obese *versus* Obese + AM251: *t* = 2.761, *p* = 0.0172, df = 12) (Figures 8C,D). Taken together, these results show changes in CB_1_ receptor-dependent long-term plasticity at IL excitatory synapses of obese Zucker rats.

### The functional response of CB_1_ receptors to WIN 55,212-2 is increased in membranes of the medial prefrontal cortex of obese Zucker rats

**FIGURE 9:**
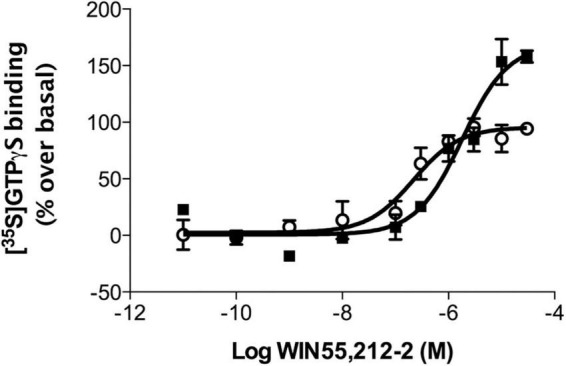
WIN 55,212-2-stimulated [^35^S]GTPγS binding in rat brain cortical membranes obtained from medial prefrontal cortex of lean (°) and obese (∙) Zucker rats. Data are mean ± SEM from three different experiments performed in duplicate. Data were normalized using the Bottom and Top values of WIN55,212-2 stimulated [^35^S]GTPγS binding obtained in lean Zucker rats.

To determine whether the differences in CB_1_ receptor expression were correlated with differences in receptor coupling to their cognate Gα_*i/o*_ proteins, [^35^S]GTPγS binding assays were performed in brain cortical membranes obtained from NCx, mPCx, and hippocampus of lean and obese Zucker rats. According to the results of immunoblot analysis described above, CB_1_ receptor coupling maximally stimulated by WIN 55,512-2 was increased in mPCx (but not NCx or hippocampal) membranes of obese Zucker rats (Table 2 and Figure 9). Thus, cannabinoid agonist WIN 55,212-2 stimulated [^35^S]GTPγS binding in a concentration-dependent manner in mPCx membranes obtained from both lean and obese Zucker. However, the maximal response (*E*_*max*_) value was 62.5% higher (*t* = 8.72, *P* = 0.0129, df = 4) and the *EC*_50_ 4.6 times more in mPCx membranes from obese Zucker relative lean rats (*t* = 3.38, *p* = 0.0278, df = 4) (Table 2 and Figure 9).

**TABLE 2:** Computer assisted curve fitting of WIN 55,212-2-stimulated [^35^S]GTPγS to brain membranes of Zucker rats.

	Lean		Obese	
	*E*_*max*_	*EC*_50_ (μ M)	*E*_*max*_	*EC*_50_ (μ M)
Medial prefrontal cortex	100	0.3 ± 0.1	162.5 ± 9.5*	1.4 ± 0.5*
Neocortex	100	0.5 ± 0.1	96.9 ± 9.3	0.9 ± 0.1
Hippocampus	100	0.7 ± 0.1	90.7 ± 1.9	0.6 ± 0.1

*E_max_* values are expressed as percent over lean. Data are means ± SEM (*n* = 3). Significance of difference from the corresponding values in lean counterparts (*P < 0.05) is shown.

### Gα_*i*2_ subunit is down-regulated in cortical brain membranes of obese Zucker rats

**FIGURE 10:**
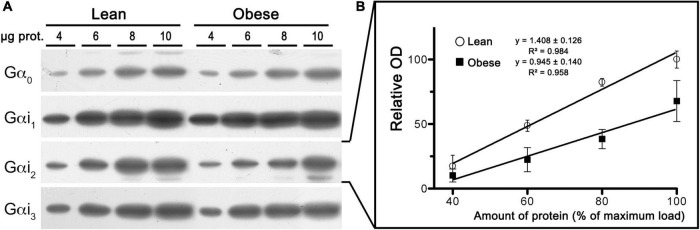
Expression analysis of Gα_*i/o*_ subunits in crude membranes of mPCx from lean and obese Zucker rats. **(A)** Representative images of immunoblots using specific antibodies against different Gα_*i/o*_ subunits. Increasing amounts of mPCx membrane samples from lean and obese Zucker rats. **(B)** Linear regression analysis comparing the relative optical density measurements of Gα_*i*2_-immunoreactivity in crude membrane samples of mPCx from lean (°) and obese (∙) Zucker rats. The correlation coefficients are provided. Data presented are means ± SEM of three independent experiments.

To test whether a compromised CB_1_-Gα_*i/o*_ protein coupling might explain the lower potency (EC_50_ values) of WIN 55,212-2 to stimulate [^35^S]GTPγS binding in mPCx of the obese Zucker rats, the expression of Gα_*i/o*_ subunits were analyzed using isoform-specific antibodies. As shown in Figure 10, linear regression analysis of immunoblots using increasing amounts of crude membranes from mPCx of lean and obese Zucker rats revealed a marked decrease in the expression of Gα_*i*2_ subunit in obese Zucker rats [slope differences, *F*_(1_,_18)_ = 4.807, *p* = 0.0417]. The ratio of slopes between obese and lean Zucker rats showed that expression of Gα_*i*2_ decreased by 33% in the mPCx of obese Zucker rats.

### Endogenous 2-AG levels are increased in several cortical areas of obese Zucker rats

**FIGURE 11:**
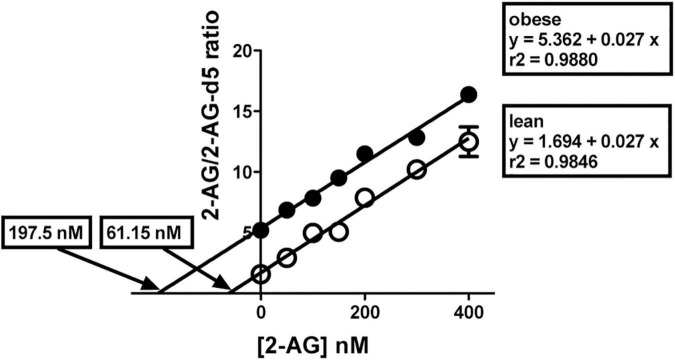
2-AG levels in brain cortical regions. Representative illustration of the method of isotopic dilution combined with standard addition techniques to determine endogenous 2-AG levels in brain cortex. Three separate experiments, each with 21 subsamples (aliquots of the brain cortical homogenate), were performed in triplicate. Prior to addition of ethylacetate/hexane for extraction, 2-AG-d_5_ and 1-AG-d_5_ (both, 100 nM), and 2-AG standard at varying amounts (0, 50, 100, 150, 200, 300, and 400 nM) were added to the subsamples. Results for the normalized signal strength for 2-AG/2-AG-d_5_ from lean (°) and obese Zucker prefrontal cortex (∙) are presented with individual best fit lines. The lines are essentially parallel but have different intercepts. Each data point is the mean ± SD of a triplicate in one experimental assay. Three independent assays were performed to obtain data shown in Table 3.

We next quantified 2-AG in mPCx and anterior and posterior NCx samples. 2-AG levels (nmol/g tissue) were between 2- and 4-fold higher in the three cortical areas (highest in mPCx [*t* = 21.74, *p* < 0.0001, df = 4] followed by the anterior [*t* = 14.16, *p* < 0.0001, df = 4] and posterior NCx [*t* = 14.40, *P* = 0.0001, df = 4]) of obese *versus* lean rat (Figure 11 and Table 3). Furthermore, linear regression analysis of immunoblots using increasing amounts of crude membranes from mPCx of lean and obese Zucker rats showed no differences in PLC-β_1_ expression between the two phenotypes (Supplementary Figure 4), ruling out the possibility that a PLC-β_1_ increase in the mPCx of the obese Zucker rat could be responsible for the drastic increase in 2-AG.

**TABLE 3:** 2-AG levels (nmol/g tissue) in individual brain cortical regions as depicted in Figure 1.

	Lean	Obese
Medial prefrontal cortex	11.2 ± 0.7	41.4 ± 1.2*
Anterior neocortex	11.0 ± 0.5	36.1 ± 1.7*
Posterior neocortex	10.3 ± 0.3	22.6 ± 0.8*

Data are expressed as means ± SEM (*n* = 3). Significance of difference from the corresponding values in lean counterparts (*P < 0.05) is shown.

## Discussion

This work provides the first in-depth analysis of the changes that take place in the expression of the CB_1_ cannabinoid receptor in the mPCx of the obese Zucker rat. Our report demonstrates a selective increase in CB_1_-expressing excitatory terminals of PL and IL of the mPCx in the obese Zucker rat. In addition, we report changes in the expression of CB_1_ receptors and Gα_*i*2_ subunits in mPCx membranes from obese Zucker rats, along with alterations in functional CB_1_-G_*i/o*_ coupling. Furthermore, this study shows that the endogenous 2-AG is increased in this brain region. Importantly, the results presented here are in good agreement with previous studies characterizing the expression of CB1 receptors in the obese Zucker rat (Thanos et al., 2008b; Zarate et al., 2008a). Taking into consideration past and present results, there is robust evidence of an up-regulation of CB_1_ receptors in the frontal cortical and related limbic areas of the obese Zucker rat. However, it should be noted that the changes in the expression of the CB_1_ receptor observed, rather than a general phenomenon affecting all cortical areas, are limited to selective regions (e.g., mPCx). In fact, our results show that CB_1_ receptors and their coupling to G_*i/o*_ proteins are increased in medial prefrontal cortical membranes of obese Zucker rats compared to lean subjects, but not in neocortical and hippocampal membranes. Moreover, no changes in CB_1_-immunostaining intensity were observed in the primary motor cortex and hippocampus. Even so, because samples used for the biochemical analysis of CB_1_ receptor expression encompass multiple anatomically distinct subdivisions of neocortex and hippocampus, it is conceivable that the expression of the CB_1_ receptor may also be altered in neocortical areas not tested separately. In fact, our previous results (Zarate et al., 2008a) showed that CB_1_ staining intensity is increased in the frontal, cingulate, and piriform cortex, but not in the parietal and temporal cortex. Nonetheless, in the study by Zarate et al. (2008a), CB_1_-immunoreactivity was observed not only at axon terminals as we describe here and is widely accepted, but also at controversial somatodendritic locations. In this regard, our recent report addressing the specificity of anti-CB_1_ receptor antibodies (Echeazarra et al., 2021) showed that the CB1-Go-Af450 antibody used here produces CB_1_-specific immunostaining circumscribed to axonal fibers and presynaptic-like puncta, whereas the goat polyclonal K15 antibody used for immunohistochemical staining in the study by Zarate et al. (2008a) produces non-specific somatodendritic signals. In addition, the CB1-Go-Af450 antibody has proven to be adequate to describe the ultrastructural distribution of CB_1_ receptors (Lafourcade et al., 2007; Peñasco et al., 2020; Bonilla-Del Río et al., 2021; Egaña-Huguet et al., 2021). In conclusion, the antibody used here has been rigorously tested and validated for specificity in a variety of applications, which is essential for a correct interpretation of the results. Indeed, cross-reactivity of antibodies with unwanted proteins is one of the main causes of the lack of reproducibility between studies (Saper, 2005; Voskuil, 2014; Taatjes and Roth, 2021).

The present study also reports findings regarding the long-term synaptic plasticity in the mPCx of the lean and obese Zucker rats. Electrophysiological recordings were taken from layers II/III of the IL area, as it was in that subregion of the mPCx that we found the strongest increase in CB_1_-immunostaining in tissue sections. Although the protocol used here, consisting in prolonged stimulation at frequencies around 10 Hz, is optimal to induce eCB-eLTD in other brain regions (Lafourcade et al., 2007; Puente et al., 2011), it induced a significant CB_1_-dependent LTP of evoked fEPSPs in obese rats, whereas elicited a CB_1_-independent LTD in lean rats. The up-regulation of CB_1_ receptors at glutamatergic synapses in the mPCx of the obese Zucker rats (as demonstrated by immunogold-silver staining and electron microscopy) could underlie these observations. However, in contrast with previous data reporting a prominent role of CB_1_ receptors in LTD at the excitatory synapses of the mPCx (Lafourcade et al., 2007), here we observed that CB_1_ receptors are involved in LTP elicited in obese rats by low-frequency stimulation, but not in LTD that the same stimulus produces in lean littermates. A potential source for the discrepancy between studies may arise from differences in the chosen mPCx subregion for recordings. Thus, in the study by Lafourcade et al. (2007), CB_1_-LTD was observed at pyramidal synapses of layers V/VI of the PL area, whereas our recordings were made in layers II/III of the IL area. Another critical variable that may contribute to the difference could be the age and the type of the animals used; 8–12 weeks old rats here *versus* 4–6 weeks old mice in Lafourcade’s study.

Biochemical experiments intended at characterizing the levels and G_*i/o*_ coupling of CB_1_ receptors in the mPCx of the obese Zucker rats provided very consistent results. Thus, immunoblot signal intensity for CB_1_ receptor increased by 1.57-fold in membranes of obese Zucker rats compared to lean subjects, whereas WIN 55,212-2-stimulated specific binding of [^35^S]GTPγS increased by 1.62-fold. This is consistent with the ability of the WIN 55,212-2-bound CB_1_ receptor to recruit more G_*i/o*_ proteins in the mPCx of the obese Zucker rat and suggests that the availability of Gα_*i/o*_ proteins is not rate limiting. The absence of changes in the basal levels of [^35^S]GTPγS binding in membranes of the mPCx of the obese Zucker rats as compared with their lean littermates suggests that the CB_1_ receptor may be not constitutively active and producing agonist-independent receptor activation. Although results of the biochemical experiments performed here cannot discriminate signals originating from CB_1_ receptors located specifically at glutamatergic or GABAergic terminals, we presume that the increase of CB_1_-immunoreactivity and maximal efficacy of WIN 55,212-2 to stimulate [^35^S]GTPγS binding in mPCx membranes of obese rats is due to the up-regulation of CB_1_ receptors at glutamatergic terminals (as demonstrated here by electron microscopy). In the same way, we infer that the increase in the WIN 55,212-2 maximal efficacy observed in mPCx membranes is a consequence of an increased coupling of glutamatergic CB_1_ receptors to G_*i/o*_ proteins. Indeed, as noted above, the strengthening of CB_1_ receptor-mediated G_*i/o*_ signaling as a result of its up-regulation at glutamatergic synapses can explain the emergence of CB_1_-dependent LTP of evoked fEPSPs in the mPCx of the obese Zucker rats. The observed up-regulation of CB_1_ receptor expression concomitantly with an increase in the efficacy of agonist-stimulated CB_1_ receptor coupling to G_*i/o*_ proteins reinforces the classical idea that CB_1_ receptor density is the main element determining the magnitude of responses to eCBs. In fact, we have recently demonstrated that the extent of the coupling of CB_1_ receptors to G_*i/o*_ proteins in frontal cortical and hippocampal synaptosomes of specific (glutamatergic and GABAergic) neurotransmitter phenotypes correlates with the abundance of CB_1_ receptors (Saumell-Esnaola et al., 2021). However, despite the traditional receptor theory would not predict a decrease in potency for CB_1_ agonists under conditions of increased CB_1_ receptor levels, WIN 55,212-2 was 4.6-fold less potent in stimulating [^35^S]GTPγS binding in the mPCx membranes of the obese Zucker rat than in those from lean littermates. Also, the possibility that changes in the transduction machinery downstream of the CB_1_ receptor could account for the observed phenomenon has to be considered. In this sense, our results show for the first time that the Gα_*i*2_ subunit is down-regulated in the mPCx of obese rats. However, the situation bears comparison with previously published results in membranes of hepatocytes and adipocytes of obese Zucker rats (Bushfield et al., 1990; Strassheim et al., 1991), hepatocytes from obese (ob/ob) mice (McFarlane-Anderson et al., 1992) and fat cells obtained from obese human subjects (Ohisalo and Milligan, 1989). The results indicate a lower expression and function of Gα_*i/o*_ subunits in obese individuals leading to a reduction of the potency of p[NH]ppG, a non-hydrolyzable GTP analog, to inhibit forskolin-stimulated adenylate cyclase activity. Moreover, Bushfield et al. (1990) concluded that their results may be explained by an increased protein kinase C activity in hepatocytes of obese Zucker rats leading to phosphorylation-induced inactivation of Gα_*i*2_ proteins. The possibility that this phenomenon could be present in the brains of obese animals only has been evaluated in whole brain homogenates of obese (ob/ob) mice with negative results (McFarlane-Anderson et al., 1992). However, as in our present study, it is possible that a separate analysis of discrete areas of the brain could find differences between the obese and lean phenotypes. In summary, one may wonder whether a decreased availability of Gα_*i*2_ proteins could plausibly explain the potency loss of WIN 55,212-2 observed in obese rats. This possibility claims that WIN 55,212-2-bound CB_1_ receptor activates multiple G_*i/o*_ proteins simultaneously, and both the efficacy and potency of the agonist to activate individual Gα_*i/o*_ subunits may vary considerably. In this context, the CB_1_ receptor provides a key example of ligand-directed GPCR functional selectivity that extends even to its actions on the classic pertussis toxin-sensitive G_*i/o*_ signaling (Hudson et al., 2010). Thus, several laboratories have found agonist-specific differences in potency and/or intrinsic activity of the CB_1_ receptor toward different Gα_*i/o*_ subtypes (Glass and Northup, 1999; Prather et al., 2000; Mukhopadhyay and Howlett, 2005). For instance, stimulation of CB_1_ receptors by WIN 55,212-2 results in the activation of a distinct pattern of at least five different Gα_*i/o*_ subunits in several brain regions and, most importantly, the concentration of WIN 55,212-2 required to half maximally activate individual G proteins in the cerebellum varied over a 30-fold range for different Gα_*i/o*_ subunits (Prather et al., 2000). Therefore, we are tempted to speculate that the rightward shift in the concentration-response curve for WIN55,212-2-stimulated [^35^S]GTPγS binding in mPCx membranes of obese Zucker rats is related to the recruitment of a population of Gα_*i/o*_ proteins in which the contribution of Gα_*i*2_ has been reduced and has been compensated by other Gα_*i/o*_ proteins with lower affinity for the WIN55,212-2-bound CB_1_ receptor. A detailed pharmacological analysis comparing the potencies and efficacies of various cannabinoid agonists, and especially the endogenous agonist 2-AG, in activating individual Gα_*i/o*_ proteins should help address this question and is currently an experimental objective in our laboratory.

Our results also show that 2-AG levels in cortical areas of obese Zucker rats are increased by 2- to 4-fold as compared to their lean littermates. Previous results also showed and increased accumulation (2-fold) of 2-AG in the hypothalamus of obese Zucker rats with respect to their lean controls (di Marzo et al., 2001). Moreover, according to our present and previous data (di Marzo et al., 2001), both the pattern of the regional differences in the levels of 2-AG (i.e., hypothalamus *versus* frontal cortex) and the absolute hypothalamic and cortical 2-AG levels (nmoles/g brain tissue) in lean Zucker rats are similar to those reported in Sprague–Dawley and Wistar rats (Bisogno et al., 1999; di Marzo et al., 2001; Richardson et al., 2007). The biosynthesis of 2-AG involves only one family of lipids, the sn-1-acyl-2-arachidonoylglycerols (DAGs), which are mostly produced by the hydrolysis of phosphoinositides (PI) *via* PI-specific PLC activity. DAGs are used as biosynthetic precursors of 2-AG through the action of either of the two sn-2-selective DAG lipases DAGLα and DAGLβ (di Marzo and de Petrocellis, 2012). With respect to the inactivation of 2-AG, the prominent role of monoacylglycerol lipase (MAGL) has been unequivocally established (Dinh et al., 2002, 2004). Although the mechanism of increased 2-AG levels in the prefrontal cortex of the obese Zucker rats reported here is still unclear increased levels of both DAGL and MAGL activities were reported in the spinal cord of obese Zucker rats (Farooqui et al., 1987). Additionally, recently it has been defined a novel mechanism for DAGLα regulation by calcium/calmodulin-dependent protein kinase II (CaMKII) (Shonesy et al., 2013). The authors show that CaMKIIα binds to and phosphorylates DAGLα, and inhibits 2-AG synthesis *in vitro*. Moreover, behavioral studies indicate that CaMKIIα is a negative regulator of 2-AG signaling *in vivo* (Shonesy et al., 2013). Interestingly, down-regulation of phosphorylated and total CaMKII has been reported in the hippocampal CA1 region of obese Zucker rats (Alzoubi et al., 2005) and in animal models of chronic psychosocial stress (Gerges et al., 2004).

The data discussed so far support the hypothesis that the obese Zucker rats may represent a preclinical model of genetic vulnerability to obesity, due to hyperactivity of endocannabinoid signaling in the mPCx that would shift the E/I balance, thus increasing the inhibitory tone in this brain area. Previous studies using conditional mutant mice lacking CB_1_ receptors either in cortical glutamatergic neurons (Glu-*CB*_1_-KO) or GABAergic neurons (GABA-*CB*_1_-KO) (Monory et al., 2006, 2007) revealed some clues to explain how CB_1_ receptors located in excitatory and inhibitory terminals of mPCx could contribute to the behavioral phenotype of the obese Zucker rat. When the conditional mutant mice Glu-*CB*_1_-KO and GABA-*CB*_1_-KO were subjected to experimental protocols that allowed for assessing food consumption, the opposite phenotypes appeared clearly (Lafenêtre et al., 2009; Bellocchio et al., 2010). Glu-*CB*_1_-KO mice were hypophagic, and the phenotype was pharmacologically related to the glutamate actions on NMDA receptors. Conversely, GABA-*CB*_1_-KO mice were more hyperphagic than their wild-type littermates, and the phenotype was a consequence of an increased GABA-A receptor activation (Bellocchio et al., 2010). Moreover, when the experimental protocol was designed to assess the behavioral inhibition component of impulsivity (Pattij et al., 2007), the results suggested that the deletion of CB_1_ receptors in glutamatergic neurons leads to an increased behavioral inhibition in the approach to novel palatable food, whereas GABA-*CB*_1_-KO mice did not show any safety behavior toward novel food, and immediately consumed the maximal amount (Lafenêtre et al., 2009). As discussed by the authors (Lafenêtre et al., 2009; Bellocchio et al., 2010), a reduction of glutamatergic transmission through activation of the endocannabinoid system would decrease behavioral inhibition, and thereby increase impulsivity in the approach to food. In support of these findings, most recent data demonstrated that the lack of CB_1_ receptors in dorsal telencephalic glutamatergic neurons abrogated the overconsumption of palatable food and the development of obesity (Ruiz de Azua et al., 2021) by promoting a resilient phenotype to food addiction (Domingo-Rodriguez et al., 2020). Consistently, in standard rodent animals, the systemic blockade of glutamatergic transmission leads to an increase in impulsive behavior (Mirjana et al., 2004; Floresco et al., 2008).

One interesting question arising from the results presented here is related to the potential impact that the hyperactivity of CB_1_ receptor signaling at the glutamatergic terminals of the PL and IL subregions of mPCx could have on the firing activity of subcortical monoaminergic neurons (i.e., ventral tegmental area-VTA). It is in this frame of reasoning, it must be kept in mind that both the cell body of the mesolimbic dopaminergic neurons and their terminal projection areas (i.e., *nucleus accumbens*, NAcc) are innervated by glutamatergic afferents from the prefrontal cortex (Sesack et al., 1989; Takagishi and Chiba, 1991; Berendse et al., 1992). Although the mechanisms and pathways by which mPCx modulates VTA dopamine neurons projecting to the NAcc need to be clarified, previous results have demonstrated that direct glutamate injected into the mPCx selectively increased burst firing of single dopamine cells in the VTA area and enhanced the release of dopamine from nerve terminals in the NAcc (Murase et al., 1993). Moreover, the specific pharmacological blockade of NMDA receptors in the mPCx is able to produce the neurochemical and motor changes associated with the dysfunction of the corticostriatal circuit, including an increase in dopamine and acetylcholine release in the NAcc and also an increase in motor activity (del Arco et al., 2008). Importantly, the chemogenetic inhibition of the glutamatergic neuronal activity of a specific prelimbic-*nucleus accumbens* pathway, which is indeed also under the control of CB_1_ receptors, induces compulsive food seeking (Domingo-Rodriguez et al., 2020). Although we focus on the role of the prefrontal cortex on inhibitory control and impulsivity (Volkow et al., 2008a,b; Boeka and Lokken, 2011), we are aware that the functions of the prefrontal cortex that contribute to obesity could be those that are sensitive to modulation by the neurotransmitter dopamine *via* striatal prefrontal pathways. Clinical neuroimaging studies in obese subjects have provided evidence that the association of striatal D_2_ dopamine receptors with inhibitory control and with impulsivity is mediated in part by their modulation of prefrontal regions (Epstein et al., 2007; Volkow et al., 2008b). Similarly, preclinical studies have shown that animals with low striatal D_2_ dopamine receptor levels, including the obese Zucker rat, are more impulsive than their littermates with higher D_2_ dopamine receptor levels (Dalley et al., 2007; Thanos et al., 2008a; Boomhower et al., 2013). Finally, additional studies are needed to clarify whether the observed up-regulation of CB_1_ receptors at glutamatergic terminals of the PL and IL subregions of the medial prefrontal cortex reported here serves an adaptive purpose, as it has been demonstrated in a murine model of obesity with leptin deficiency (Cristino et al., 2013). In that study, authors reported that orexigenic neurons of the lateral hypothalamic area (LH) of the *ob/ob* mice undergo a shift from predominant control by CB_1_-expressing excitatory to CB_1_-expressing inhibitory inputs, a phenomenon that was partly reversed by leptin administration *via* the mTOR branch of the leptin receptor signaling cascade (Cota et al., 2006).

In conclusion, the results of the present study show that the Zucker lines (lean and obese) represent two homogeneous populations that can be used to test the hypothesis on the involvement of hyperactivity of the medial prefrontal endocannabinoid system at glutamatergic terminals in the individual vulnerability to obesity.

## Data availability statement

The original contributions presented in this study are included in the article/Supplementary material. Further inquiries can be directed to the corresponding author.

## Ethics statement

The animal study was reviewed and approved by Committee of Ethics for Animal Welfare of the University of the Basque Country (Ref. CEBA/199/2011/GARCIA DEL CAÑO).

## Author contributions

LE, SB, GG, IB-D, JE-H, NP, XA, MM, ML, IG-B, MS-E, MG, and JS performed or contributed to the experiments. SB, GG, IB-D, JE-H, NP, and MG performed the analyses. GG, PG, MG, and JS designed the study and drafted the first manuscript. All authors reviewed and approved the final manuscript.

## Abbreviations

2-AG: 2-arachidonoylglycerol
[^35^S]GTPγS: guanosine 5′-o-(3-[^35^S]thio-triphosphate
AC: anterior cingulate cortex
CaMKII: calcium/calmodulin-dependent protein kinase II
CB_1_: receptor (cannabinoid type 1 receptor)
DAG: diacylglycerol
DAGL: diacylglycerol lipase
DG: *Dentate gyrus*
eCB-eLTD: endocannabinoid-mediated excitatory long-term depression
EGTA: ethylene glycol bis (2-aminoethyl ether) tetraacetic acid
fEPSP: field excitatory postsynaptic potential
IL: infralimbic cortex
LC/MS-MS: liquid chromatography and mass spectrometry
LFS: low-frequency stimulation
LH: lateral hypothalamic area
LTD: long-term depression
LTP: long-term potentiation
MAGL: monoacylglycerol lipase
Mop: primary motor cortex
mPCx: medial prefrontal cortex
NAcc: *nucleus accumbens*
NCx: neocortex
PI: phosphoinositides
PLC-β_1_: phospholipase C-β_1_
PMSF: phenylmethylsulfonyl fluoride
PTX: picrotoxin
PL: prelimbic cortex
VTA: ventral tegmental area.
