# Supplementary material for: Up-regulation of CB1 cannabinoid receptors located at glutamatergic terminals in the medial prefrontal cortex of the obese Zucker rat
Source: Front Neuroanat. 2022 Oct 18;16:1004702. doi: 10.3389/fnana.2022.1004702 (PMC9623818; doi:10.3389/fnana.2022.1004702)
Supplement: Supplementary file 1 [file Data_Sheet_1.PDF]

Supplementary Material

SUPPLEMENTARY RESULTS

Distribution of CB_1-immunostaining

To determine consistency of the immunolabelling pattern with that described in previous studies (Egertová and Elphick, 2000; Bodor et al., 2005; Deshmukh et al., 2007), apart from the analysis performed in NCx and mPCx as described in the main manuscript, we examined the immunostaining pattern in the hippocampal formation and several subcortical regions of the lean Zucker rat. CB_1-immunostaining completely delineated the various regions and layers of the Ammon's horn and dentate gyrus (DG). A row of dense immunolabelling was observed throughout the *stratum pyramidale* (sp) of the Ammon's horn from CA1 to the most distal part of CA3 (**Supplementary Figure 3A**), being stronger in CA1 and CA2 than in CA3. Labelling in sp of CA1-CA3 was particularly dense around cell bodies surrounding of pyramidal cells, which were apparently devoid of immunoreactivity. Immunostaining was apparent in axon-like profiles of the *strata oriens* (so) and *radiatum* (sr), but considerably weaker than in sp (**Supplementary Figure 3B**). In the dentate gyrus, CB_1-immunoreactivity was highest in fibres molecular layer, which formed a much denser meshwork in the inner (In mo) than in the outer (Out mo) molecular layer. More scattered immunopositive axonal profiles were observed in the polymorph layer (po), whereas only a few fibres intermingled with unstained cell bodies of granule cells could be observed in the stratum granulosum (sg) (**Supplementary Figure 3C**). CB_1-immunopositive cells were also present in different regions of the hippocampal formation, most of them being located in so and sr of the Ammon's horn and at the border between the sg and po of DG (**Supplementary Figures 3B-C**). In the amygdaloid complex, CB_1-immunoreactivity appeared as an axonal meshwork of axon-like profiles in the lateral (LA) and basolateral (BLA) nuclei, followed by the basomedial (BMA) nucleus. Considerably weaker immunostaining was observed in the intercalated (IA) nucleus, whereas only a diffuse background was seen in the lateral part of the central nucleus of amygdala (CEAl) (**Supplementary Figure 3D**). In the basal ganglia, neuropil immunostaining was evident in the gray matter of caudate-putamen and increased gradually from the medial to lateral regions (not shown). A dense plexus of intensely immunostained fibres intermingled with unstained fascicles was observed in the lateral globus pallidus (not shown), and an oval-shaped core of particularly strong immunoreactivity delineated the boundaries of substantia nigra reticulata (SNr) (**Supplementary Figure 3E**).

SUPPLEMENTARY METHODS

Immunohistochemical staining

For immunohistochemical staining of the CB_1 receptor, brain sections from all animals (5 lean and 5 obese Zucker rats) were processed simultaneously, in a free-floating manner, with the same amount of freshly prepared solutions. After a treatment with 1% H_2O_2 in PBS for 20 min, sections were incubated at room temperature for 1 h in PBS, containing 1% serum albumin bovine (BSA; Sigma, St. Louis, MO, USA) and 1% normal rabbit serum (blocking solution). Sections were then incubated overnight at 4 ºC with an affinity purified goat polyclonal antibody raised against the C-terminal 31 amino acids (443-473) of the mouse CB_1 receptor (Ref. CB1-Go-Af450; Frontier Science Co. Ltd, Hokkaido, Japan), diluted at 1 µg/mL in blocking solution. After three wash steps with PBS (10 min each) at room

temperature, sections were incubated for 1 h at room temperature with an affinity purified biotinylated rabbit anti-goat IgG (Ref. BA-5000; Vector Laboratories, Burlingame, CA, USA) diluted 1:200 in blocking solution, followed by three wash steps with PBS (10 min each). Thereafter, tissue sections were incubated with avidin-biotin-peroxidase complex (Vectastain ABC Elite kit; Vector Laboratories) diluted 1:200 in PBS, washed once with PBS (10 min) and twice with 50 mM Tris-HCl, pH 7.6 (10 min each), and reacted with 0.05% 3,3'-diaminobenzidine tetrahydrochloride and 0.01% H_2O_2 in 50 mM Tris-HCl, pH 7.6. Finally, sections were thoroughly washed with PBS, mounted onto gelatin-coated slides and coverslipped using DPX (Fluka, Buchs, Switzerland).

Preembedding immunogold for electron microscopy

Vibratome sections of the mPCx from 3 lean and 3 obese Zucker rats were processed simultaneously, in a free-floating manner, with the same amount of freshly prepared solutions. Briefly, sections were preincubated, for 30 minutes at room temperature, in a blocking solution consisting of 10% bovine serum albumin -BSA-, 1% rabbit normal serum and 0.02% saponin in Tris-HCl buffered saline, pH 7.4. Then, sections were incubated for 2 days at 4ºC with the polyclonal anti-CB_1 receptor CB1-Go-Af450 antibody (Frontiers Sciences Institute), diluted at 1 µg/mL in Tris-HCl buffered saline (TBS) containing 10% BSA and 0.004% saponin. After three 10 min washes in 1% BSA/TBS, tissue sections were incubated in a secondary 1.4 nm gold-labelled rabbit anti-goat Immunoglobulin-G (Fab' fragment, 1:100, Nanoprobes Inc., Yaphank, NY, USA) in 1% BSA/TBS with 0.004% saponin for 4 h at room temperature. After washing in 1% BSA/TBS overnight at 4 °C, sections were postfixed in 1% glutaraldehyde in TBS for 10 min followed by three 10 min washes in double-distilled water. Then, gold particles were silver-intensified with a HQ Silver kit (Nanoprobes Inc., Yaphank, NY, USA) for about 12 min in the dark and sections were washed in 0.1M PB. Stained sections were osmium-intensified for 20 min with 1% OsO_4 in 0.1M PB, dehydrated in graded alcohols to propylene oxide and plastic-embedded in Epon resin 812. Ultrathin sections of 50 nm were collected on nickel mesh grids, stained with 2.5% lead citrate for 20 min and examined using a Philips EM208S electron microscope.

Semiquantitative analysis of the CB_1-immunostaining

For each OD measurement, images of all regions under analysis were acquired from two coronal sections located between bregma AP +2.80 and +2.15 (cortex) or two coronal sections located between bregma AP -3.90 and -4.45 (hippocampal formation). Due to the laminar distribution pattern of the immunostaining in the cortex, measurements were performed in layers showing the strongest CB_1-immunoreactivity (layers II-III, Va and VI). For each cortical area of interest, measurements were performed within 3 regions of interest (ROIs) of 150 x 150 μm^2 per layer and hemisphere (**Supplementary Figure 1A**), giving a total of 36 values per animal and cortical area. Measurements in the hippocampal formation were performed within one (CA2), two (dentate gyrus, DG) or three (CA1 and CA3) ROIs of 300 x 300 μm^2 encompassing stratum pyramidale (sp) and adjacent strata of CA1-CA3 or stratum granulosum (sg) and adjacent strata of DG (**Supplementary Figure 1B**), giving a total of 4 (CA2), 8 (DG) or 12 (CA1, CA3) values per animal. The background staining in each section analysed was considered as the mean OD value obtained in three ROIs of 150 x 150 μm^2, which were selected in zones of the subcortical white matter displaying the lightest staining intensity. All measurements within the 16-bit 0-65535 (black-white) range were calibrated to an arbitrary scale of 0-65535, with 0 being white and 65535 being black, and background OD was subtracted for each measurement. Finally, each OD data for a given cortical or hippocampal region was calculated as the average OD (corrected for background) obtained from all ROIs within that region. All OD values from

different cortical regions were normalized to a percentage of the mean OD obtained in the primary motor cortex (MOp) of lean Zucker rats. OD values from different hippocampal regions were normalized to a percentage of the mean OD obtained in the entire hippocampal formation of lean Zucker rats. All data were thus expressed as relative OD.

Western blotting

Sample pools from tissue homogenates of lean or obese Zucker rats were boiled in urea denaturing buffer (20 mM Tris-HCl, pH 8.0, 12% glycerol, 12% Urea, 5% dithiothreitol, 2% Sodium dodecyl sulphate -SDS-, 0.01% bromophenol blue) for 5 min. Increasing amounts of total protein from sample pools of lean and obese Zucker rats were run on the same SDS-polyacrylamide gel (SDS-PAGE) and processed in parallel. After being resolved by electrophoresis using either a Mini Protean II or a Protean II xi Cell apparatus (Both, Bio-Rad; Hercules, CA, USA), proteins were transferred to polyvinylidene fluoride (PVDF; Amersham Biosciences, Piscataway, NJ, USA) using a TransBlot transfer unit (Bio-Rad). PVDF sheets were incubated for 1 hour at room temperature in 0.2 M PBS (pH 7.5), containing 5% non-fat dry milk, 0.5% BSA and 0.2% Tween-20 (blocking solution) followed by overnight incubation in the following primary antibodies diluted in blocking solution: goat polyclonal anti-CB_1 receptor (200 ng/mL; Ref. CB1-Go-Af450; Frontier Science Co. Ltd), rabbit polyclonal anti-$G\alpha_o$ (40 ng/mL; Ref. sc-387; Santa Cruz Biotechnology, Santa Cruz, CA, USA), rabbit polyclonal anti-$G\alpha_{i1}$ (40 ng/mL; Ref. sc-391; Santa Cruz Biotechnology), rabbit polyclonal anti-$G\alpha_{i2}$ (200 ng/mL; Ref. sc-7276; Santa Cruz Biotechnology), rabbit polyclonal anti-$G\alpha_{i3}$ (5 ng/mL; Ref. sc-262; Santa Cruz Biotechnology) or mouse monoclonal anti-PLC-β_1 (32 ng/mL; Ref. 610924; BD Transduction Laboratories, San Diego, CA, USA). After three wash steps (10 min each) with 0.2 M PBS containing 0.1% Tween-20, PVDF membranes were incubated for 1 hour in horseradish peroxidase-conjugated secondary antibodies (Amersham Bioscience) in blocking solution (1:10000 dilution). Immunoreactivity was visualized by chemiluminescence (ECL system, Amersham Biosciences) following the manufacturer instructions. In all experiments, blots were subsequently incubated in stripping solution [62.5 mM Tris (pH 6.7), 2% (w/v) SDS, and 100 mM β-mercaptoethanol] for 30 min at 50ºC, washed twice with PBS-T, and reproved with anti-actin antibody (1:2000) as described above, to test whether nonspecific effects could occur in our experimental conditions.

SUPPLEMENTARY FIGURES

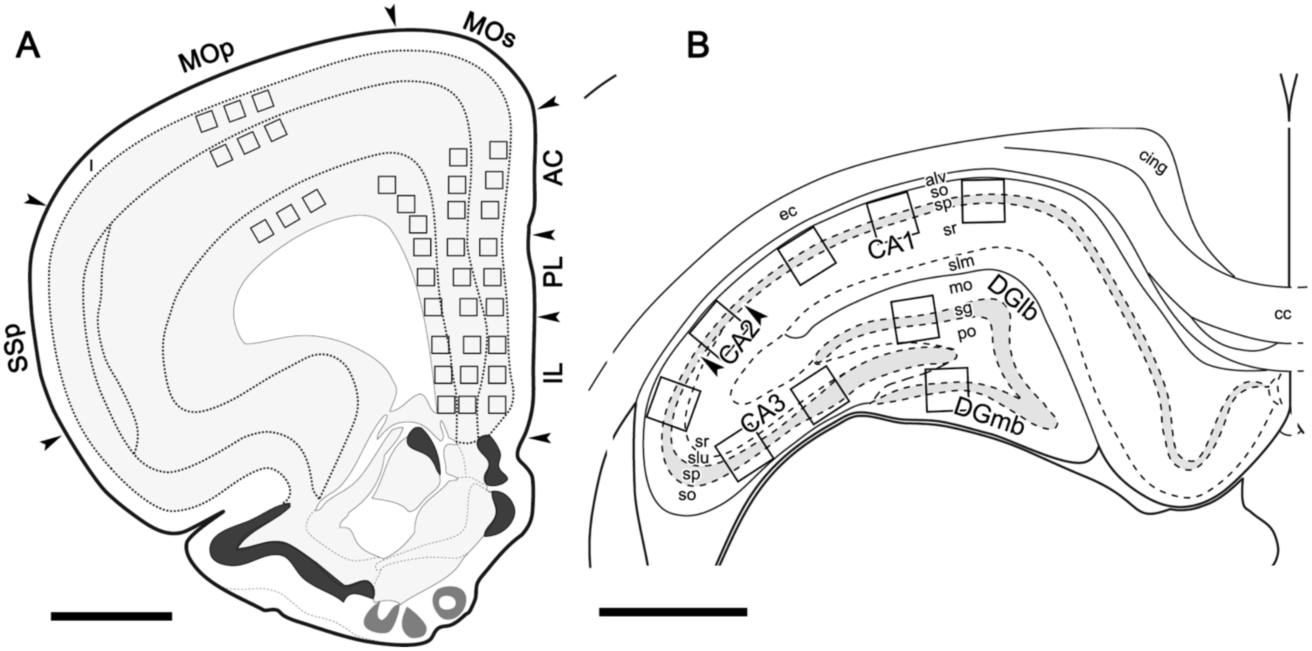

Supplementary Figure 1 (A) Schematic drawing of a coronal section of the rat brain (bregma +2.8 mm) to depict counting boxes used for measurement of CB_1-immunostaining density in primary motor cortex and subdivisions of the medial prefrontal cortex. AC, anterior cingulate cortex; IL, infralimbic cortex; MOp, primary motor cortex; MOp, secondary motor cortex; mPCx, medial prefrontal cortex; PL, prelimbic cortex; SSp, somatosensory primary cortex. **(B)** Schematic drawing of a coronal section of the rat hippocampus (bregma -4.20 mm) to depict counting boxes used for measurement of CB_1-immunostaining density in subdivision of the Ammon's horn and dentate gyrus. Alv, *alveus*; CA1-3, fields CA1-3 of the Ammon's horn; cc, corpus callosum; cing, *cingulum* bundle; DGlb, lateral blade of dentate gyrus; DGmb, medial blade of dentate gyrus; ec, external capsule; Hipp., hippocampal formation; mo, molecular layer of dentate gyrus; po, polymorph layer of dentate gyrus; sg, granular layer of *dentate gyrus*; slm, *stratum lacunosum-moleculare*; slu, *stratum lucidum*; so, *stratum oriens*; sp, *stratum pyramidale*; sr, *stratum radiatum*. The template used in both A and B were redrawn from Guillery (1993). Scale bars = 1 mm.

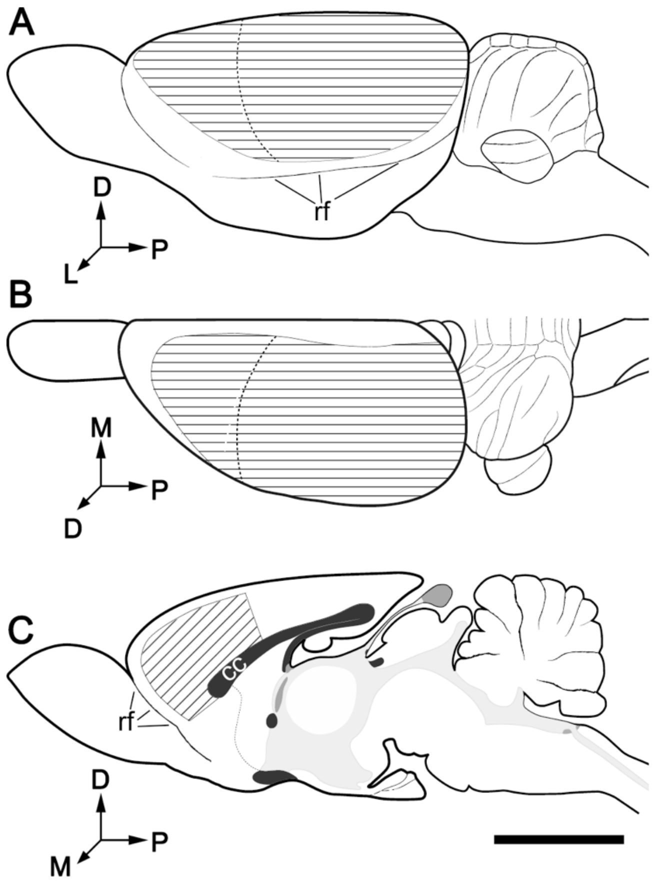

Supplementary Figure 2. Schematic drawings of the lateral **(A)**, dorsal **(B)** and medial **(C)** views of the rat brain to depict the approximate lines of dissection of cortical regions intended for Western blot and LC-MS analyses. The areas filled with horizontal lines, oblique lines and dots correspond to neocortex and medial prefrontal cortex, respectively. The midline, corpus callosum (cc), and rhinal fissure (rf) were used as anatomical landmarks for tissue dissection. The dashed line in A-B corresponds to the approximate dissection level to obtain samples of anterior and posterior neocortex for LC-MS. D, dorsal; L, lateral; M, medial; P, posterior. The template used in C was redrawn from Guillery (1993). Scale bar = 5 mm.

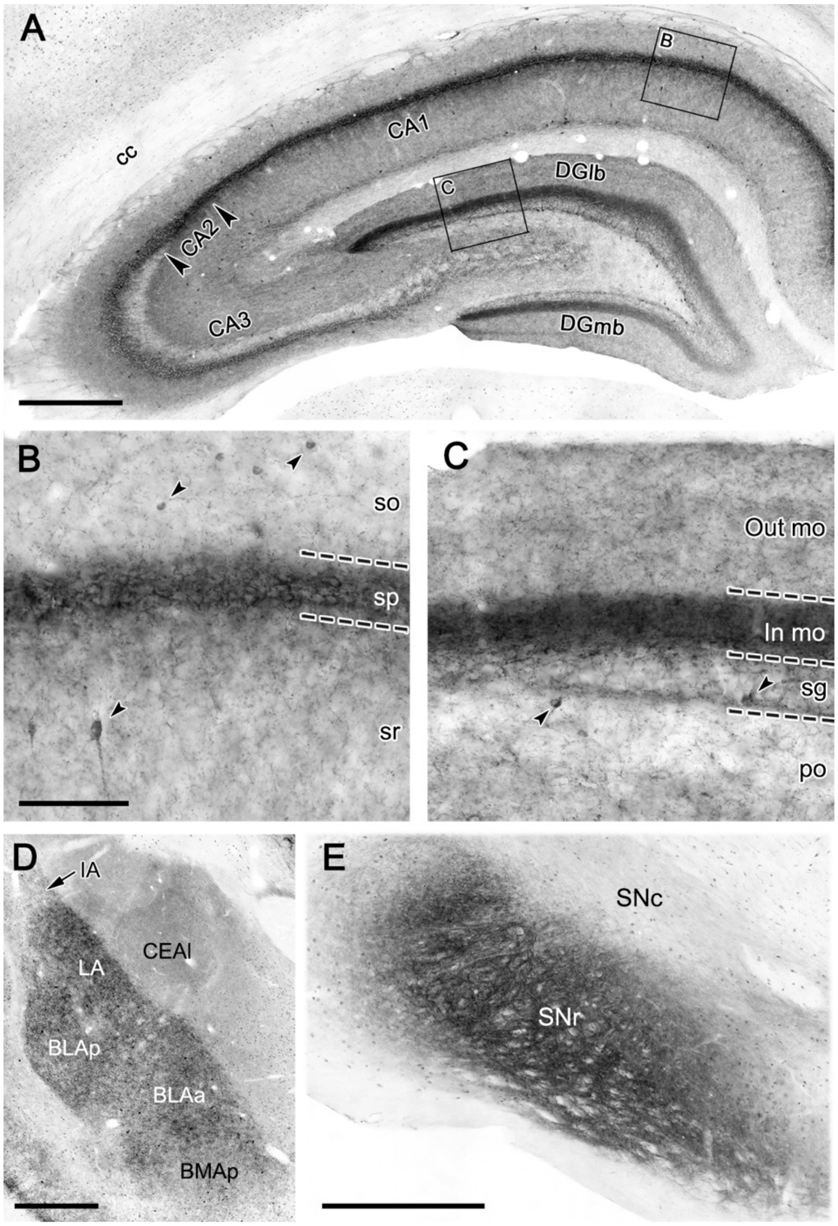

Supplementary Figure 3 CB_1-immunohistochemical staining pattern in representative regions of the brain of the lean Zucker rat. **(A)** Low magnification micrograph showing CB_1-immunostaining in the hippocampal formation. **(B,C)** Higher magnification micrographs at the level of the CA1 field of the Ammon's horn (B) and lateral blade of DG (C), corresponding to areas framed in A. **(D)** Low power micrograph showing the distribution of CB_1-immunostaining in the amygdaloid complex. Arrowheads indicate CB_1-immunopositive neuronal cell bodies. **(E)** Panoramic view of substantia nigra immunostained for CB_1. BLAa, basolateral nucleus of amygdala, anterior part; BLAp, basolateral nucleus of amygdala, posterior part; BMAp, basomedial nucleus of amygdala, posterior part; CA1-3, fields CA1-3 of the Ammon's horn; cc, corpus callosum; CEAl, central nucleus of amygdala, lateral part; DGlb, lateral blade of DG; DGmb, medial blade of DG; IA, intercalated nucleus of amygdala; In mo, inner molecular layer of DG; LA, lateral nucleus of amygdala; Out mo, outer molecular layer of DG; po, polymorph layer of DG; sg, stratum granulosum of DG; SNc, substantia nigra compacta; SNr, substantia nigra reticulata; so, stratum oriens; sp, stratum pyramidale; sr, stratum radiatum. Scale bars = 500 µm in A, D and E; 100 µm in B (applies to B-C).

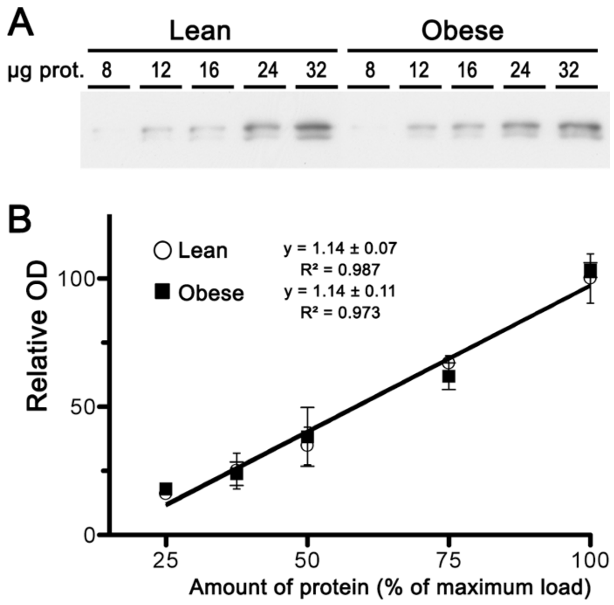

Supplementary Figure 4. Expression analysis of PLC-β1 in samples of crude membranes from medial prefrontal cortex of lean and obese Zucker rats**.** **(A)** Representative image of PLC-β_1 immunoblot using increasing amounts of total protein in crude membranes from medial prefrontal cortex of lean and obese Zucker rats. **(B)** Linear regression analyses of the relative optical density measurements of PLC-β_1 immunoreactivity obtained in crude membrane samples from medial prefrontal cortex of lean (○) and obese (●) Zucker rats. The correlation coefficients are provided. Data presented are means ± SEM of 3 independent experiments.

SUPPLEMENTARY REFERENCES

Bodor, Á. L., Katona, I., Nyíri, G., Mackie, K., Ledent, C., Hájos, N., et al. (2005). Endocannabinoid signaling in rat somatosensory cortex: Laminar differences and involvement of specific interneuron types. *Journal of Neuroscience* 25, 6845–6856. doi: 10.1523/JNEUROSCI.0442-05.2005.

Deshmukh, S., Onozuka, K., Bender, K. J., Bender, V. A., Lutz, B., Mackie, K., et al. (2007). Postnatal development of cannabinoid receptor type 1 expression in rodent somatosensory cortex. *Neuroscience* 145, 279–287. doi: 10.1016/j.neuroscience.2006.11.033.

Egertová, M., and Elphick, M. R. (2000). Localisation of cannabinoid receptors in the rat brain using antibodies to the intracellular C-terminal tail of CB1. *Journal of Comparative Neurology* 422, 159–171. doi: 10.1002/(SICI)1096-9861(20000626)422:2<159:AID-CNE1>3.0.CO;2-1.

Guillery, R. (1993). Brain maps: structure of the rat brain: by L.W. Swanson, Elsevier, 1992. Dfl.390.00 (vii + 240 pages) ISBN 0 444 81414 0. *Trends in Neurosciences* 16, 293–294. doi: https://doi.org/10.1016/0166-2236(93)90187-Q.
